# Personalized, disease-stage specific, rapid identification of immunosuppression in sepsis

**DOI:** 10.3389/fimmu.2024.1430972

**Published:** 2024-10-29

**Authors:** Theodora Pappa, Ariel L. Rivas, Michelle J. Iandiorio, Almira L. Hoogesteijn, Jeanne M. Fair, Andrea Paola Rojas Gil, Angeliki R. Burriel, Pantelis G. Bagos, Stylianos Chatzipanagiotou, Anastasios Ioannidis

**Affiliations:** ^1^ Laboratory of Basic Health Sciences, Department of Nursing, Faculty of Health Sciences, University of Peloponnese, Tripoli, Greece; ^2^ Center for Global Health-Division of Infectious Diseases, School of Medicine, University of New Mexico, Albuquerque, NM, United States; ^3^ Department of Internal Medicine, School of Medicine, University of New Mexico, Albuquerque, NM, United States; ^4^ Department of Human Ecology, CINVESTAV, Merida, YUC, Mexico; ^5^ Los Alamos National Laboratory, Los Alamos, NM, United States; ^6^ Department of Computer Science and Biomedical Informatics, University of Thessaly, Lamia, Greece; ^7^ Department of Biopathology and Clinical Microbiology, Aeginition Hospital, Medical School, National and Kapodistrian University of Athens, Athens, Greece

**Keywords:** immunology & infectious diseases, sepsis, white blood cell count (WBC), inflammation, interactions

## Abstract

**Introduction:**

Data overlapping of different biological conditions prevents personalized medical decision-making. For example, when the neutrophil percentages of surviving septic patients overlap with those of non-survivors, no individualized assessment is possible. To ameliorate this problem, an immunological method was explored in the context of sepsis.

**Methods:**

Blood leukocyte counts and relative percentages as well as the serum concentration of several proteins were investigated with 4072 longitudinal samples collected from 331 hospitalized patients classified as septic (n=286), non-septic (n=43), or not assigned (n=2). Two methodological approaches were evaluated: (i) a reductionist alternative, which analyzed variables in isolation; and (ii) a non-reductionist version, which examined interactions among six (leukocyte-, bacterial-, temporal-, personalized-, population-, and outcome-related) dimensions.

**Results:**

The reductionist approach did not distinguish outcomes: the leukocyte and serum protein data of survivors and non-survivors overlapped. In contrast, the non-reductionist alternative differentiated several data groups, of which at least one was only composed of survivors (a finding observable since hospitalization day 1). Hence, the non-reductionist approach promoted personalized medical practices: every patient classified within a subset associated with 100% survival subset was likely to survive. The non-reductionist method also revealed five inflammatory or disease-related stages (provisionally named ‘early inflammation, early immunocompetence, intermediary immuno-suppression, late immuno-suppression, or other’). Mortality data validated these labels: both ‘suppression’ subsets revealed 100% mortality, the ‘immunocompetence’ group exhibited 100% survival, while the remaining sets reported two-digit mortality percentages. While the ‘intermediary’ suppression expressed an impaired monocyte-related function, the ‘late’ suppression displayed renal-related dysfunctions, as indicated by high concentrations of urea and creatinine.

**Discussion:**

The data-driven differentiation of five data groups may foster early and non-overlapping biomedical decision-making, both upon admission and throughout their hospitalization. This approach could evaluate therapies, at personalized level, earlier. To ascertain repeatability and investigate the dynamics of the ‘other’ group, additional studies are recommended.

## 1 Introduction

Sepsis affects global health: worldwide, it causes, approximately, 11 million annual deaths (1). Early diagnosis is critical for sepsis management (2). To improve research and clinical practice, personalized assessments are essential (3). Although large research efforts have characterized this field, progress remains elusive (4–6). The accuracy of earlier definitions has been questioned because approximately half of the cases suspected to be septic do not yield positive culture results (7–10). Given the reported paucity of longitudinal studies, there have been calls for personalized methods that measure biological complexity and dynamics (5, 11–15).

New methods may focus on immunology. The immune system is the one that connects with and informs on most (if not all) biological functions and structures (16, 17). Such methods may consider: (i) the combinatorial nature of the immune system, (ii) the limitations of earlier methods, and (iii) the apparent lack of tests that rapidly distinguish immuno-suppressed patients.

‘Combinatorial’ are cognitive or communication systems in which meaning emerges afte the elementary units are structured into several levels of increasing complexity, e.g., human language. While individual *letters* lack meaning, information emerges after they are combined as *words*, *sentences*, *paragraphs*, and so on (16, 17). While there are only 26 letters in the English alphabet, the number of words is very high and the number of sentences is, apparently, infinite. Similarly, in immunology, at least 30,000 data combinations can be conceived and tested (18).

The process that generates meaning is likely to be influenced by *properties* and/or *structures*. While, in human language, it is well understood that *data* alone do not induce meaning and, consequently, *information* is needed (which should then be structured as *knowledge* and, finally, converted into *decisions* or *wisdom*, i.e., the ‘*D-I-K-W* pyramid’); the properties and structures of the immunological combinatorial system are poorly known (19).

Synergy and pleiotropy are exceptions to the previous statement. They illustrate the relevance of immunological combinations (20, 21). While the use of two words may suggest separate concepts, they are just two expressions of the same process. That is so because the same elements that, combined, can generate more or larger effects than the sum of their individual actions (synergy), when they act alone, they can perform multiple functions (pleiotropy).

Cytokines and leukocytes also possess combinatorial functions (22). A single cell type can induce not only different but even opposite functions. For example, monocytes can foster or destroy neutrophil function. To decipher which alternative applies to a specific case, the temporal phase (early vs. resolution) of the inflammatory process should be estimated –a question that, to be answered, requires bio-temporal knowledge (23).

Therefore, to obtain meaning in immunology, combinatorial perspectives should be examined over time. While they have been estimated in several diseases, including COVID-19 and hantavirus (24, 25), *sepsis-related inflammatory stages have not yet been explored.*

New methods should also prevent the limitations of earlier approaches. Because biological effects may not be predicted from their apparent causes (14, 15), prognosis is problematic. For example, virulence (a system-level property) cannot be predicted from data on isolated variables, such as virulence factors (26). Hence, the properties of complex biological systems are not reducible to putative causes (17). To address such issues, biomedical data analysis should consider, at least: (i) the compositional nature of immunological data, (ii) non-normality, (iii) data overlapping, and (iv) the limitations of artificial intelligence.

Compositional data are those in which relationships among two or more variables are key (and, therefore, should be analyzed with ratios), while counts lack meaning. Numerous biological subsystems are compositional, including the microbiome (27). Because immunology is also compositional, standard statistical tests cannot analyze immunology-related correlations (28).

Because immunological data may exhibit non-normality (29), classical statistical methods do not always apply. In addition, methods that emphasize populations −where *n*>1, which are described with averages or intervals− do not apply to personalized medicine –where *n*=1 (30).

One major example of the problem generated when population-oriented approaches are used to analyze individuals is what here is named *data overlapping*. That phrase refers to overlapping intervals of different biomedical conditions (e.g., survival and non-survival). In such situations, no personalized medical inference –no individualized discrimination– is possible even when population-related averages reach statistically significant differences (31).

Recently, artificial intelligence (AI) has been applied in sepsis (32). AI methods are classified into two (‘white box’ and ‘black box’) varieties (33, 34). While ‘white box’ approaches are transparent, ‘black box’ ones are not. These two varieties also differ in their sequences: ‘white box’ approaches are *bottom-up* (they start identifying the variables to be investigated), while ‘black box’ perspectives are *top-down* (they are data-driven, lacking a pre-established theory on what they may find, why or how (35). Hence, a conundrum: while ‘white box’ approaches may fail to inform more or better, ‘black box’ alternatives may be invalid (36).

New methods should also focus on immuno-suppression −a condition probably reversed if treated with immuno-modulators (37, 38). Unfortunately, immuno-suppressed patients are not rapidly detected (39, 40). For such a task, reductionist (self-limited) tests are not indicated when three or more alternatives exist. One example of a reductionist approach is to equal immuno-suppression with lymphopenia. While lymphopenia and low monocyte human leukocyte antigen-DR can identify immunosuppression, data overlapping prevents differentiating survivors from non-survivors in the first two hospitalization days (41). While monocyte-mediated suppression is detectable with single-cell RNA sequencing, this is a three-day long test (42).

Yet, combinatorial, immunology-centered methods offer a remedy to address the challenges listed above. They could start with a top-down approach and, after distinct *data patterns* are found, validate the findings with interpretable biomedical variables (43, 44).

Accordingly, a method that includes six (*personalized-, population-, immunological-, bacterial-, temporal- and outcome*-related) dimensions is here evaluated. It informs both on the temporal sequence of inflammatory phases and the outcomes (survival vs. non-survival) they are associated with. Because sepsis may include three or more immunological stages (such as no inflammation, excessive inflammation, and immuno-suppression), the new method handles non-binary (unlimited) and dynamic situations (11, 17). To prevent confounding, the novel approach is visually observable (45–47). It is also designed to capture personalized dimensions (19) which, to be unambiguously detected, require comparisons against populations. It addresses the *bottom-up−top-down* controversy as well as the ‘white box’/’black box’ AI problem (35, 36). Because it recognizes patterns *after data collection*, it prevents the bias and self-limited consequences of approaches that define research questions *before data collection.* To use available resources, the method under study operates with CBC data and is meant to conclude before culture results become available (48). This study was meant to elucidate whether an immunological method applicable in hematology can provide timely information on the possible outcome of the septic disorder so treatments, if needed, can be adjusted for the benefit of the patient.

## 2 Materials and methods

### 2.1 Individuals

A random sample of 331 patients admitted to three Greek hospitals between 2018 and 2022 (*n*=4072 temporal observations) was analyzed. Inclusion criteria involved meeting at least two of the systemic inflammatory response syndrome criteria: (i) body temperature >38°C, (ii) heart rate >90 beats/minute, (iii) tachypnea or hyperventilation (>20 breaths/minute or P_ACO2_ < 32 mm Hg at sea level), and (iv) a white blood cell count ≥12,000 or ≤4000/μl (49). Patients meeting these criteria and yielding at least one positive culture were viewed as “septic with positive blood culture” (i.e., *septic*). Patients meeting the same criteria and one or more negative culture(s) were regarded as “septic with a negative blood culture” (i.e., *non-septic*). The number of investigated patients was selected not according to statistical assumptions but demonstration of contents that could measure dynamic cell-cell interactions, e.g., at least 80% of the tested patients provided ≥ 5 temporal data points. Patients were excluded if they had a history of chronic diseases and/or did not meet two of the criteria mentioned above. Patients with zero percentage of any cell type were excluded. Blood samples were taken at admission from 31- to 87-year-old patients. Records were de-identified before analysis and 30-day in-hospital mortality was assessed. Conceived after patients died or were discharged, this study met the Declaration of Helsinki and was approved by the Scientific Committee of the Deanery of the Faculty of Human Sciences of Movement and Quality of Life of the University of Peloponnese (protocol 376/23.01.2018).

### 2.2 Laboratory methods

Human white blood cell counts and percentages, C-reactive protein (CPR), urea, creatinine and conventional blood cultures were analyzed. General blood examination utilized an automated hematology analyzer (Coulter LH 780 Analyzer, Beckman Coulter International SA, Nyon, Switzerland). Serum CPR concentration (mg/L) was measured with an automated system (BN ProSpec System, Siemens AG, Erlangen, Germany). Blood cultures were performed with the automated Bactec 9249 instrument (Becton Dickinson, NJ, USA). Blood pathogens were identified with the automated microbiology Phoenix 100 system (Becton Dickinson, NJ, USA).

### 2.3 Assessment and validation of dynamic complexity based on structured data

As described elsewhere (37), leukocyte data were partitioned into subsets with a three-step method, which consists of (i) *data expansion* −a step that increases the number of variables available for analysis, so that hidden patterns, if present, may be detected; (ii) pattern *recognition*−when, based on distinct patterns, the data are divided into subsets (*immune profiles*); and (iii) *statistical analysis* (a step that determines whether biological variables differ among *immune profiles*). In the first step, new metrics (combinations of leukocyte data) are created. They are dimensionless indicators (DIs) used as temporary guides that help detect data patterns. DIs have no biological definition and are reported with identifiers expressed in italics (e.g., *AAA*). This method was performed with a proprietary algorithm (US patent 10,429,389 B2).

### 2.4 Statistical analysis

Medians (Mann–Whitney test) and associations (chi-square test), as well as 3D plots were analyzed or created with a commercial package (Minitab Inc., State College, PA, USA).

## 3 Results

### 3.1 Preliminary validation

When all longitudinal data points were analyzed (n=4072), the mortality of patients presumed septic was statistically significantly greater than the mortality of non-septic individuals (DOI:10.6084/m9.figshare.25533595): 30.2% (or 1090/3605) vs. 10.1% (or 46/466), respectively (*p*<0.01, chi-square test). Hospitalization day-1 data (n=329) revealed a similar finding: a 4:1 septic/non-septic mortality ratio derived from a 28.3% mortality in septic patients (81/286), and 7.0% (3/43) in non-septic patients (2 non-septic patients were not tested on day 1, **Supplementary Table 1**). Thus, the validity of the criteria followed to recruit participants (which assumed greater mortality among septic than non-septic patients) was supported.

### 3.2 Unstructured data

**Figure 1:**
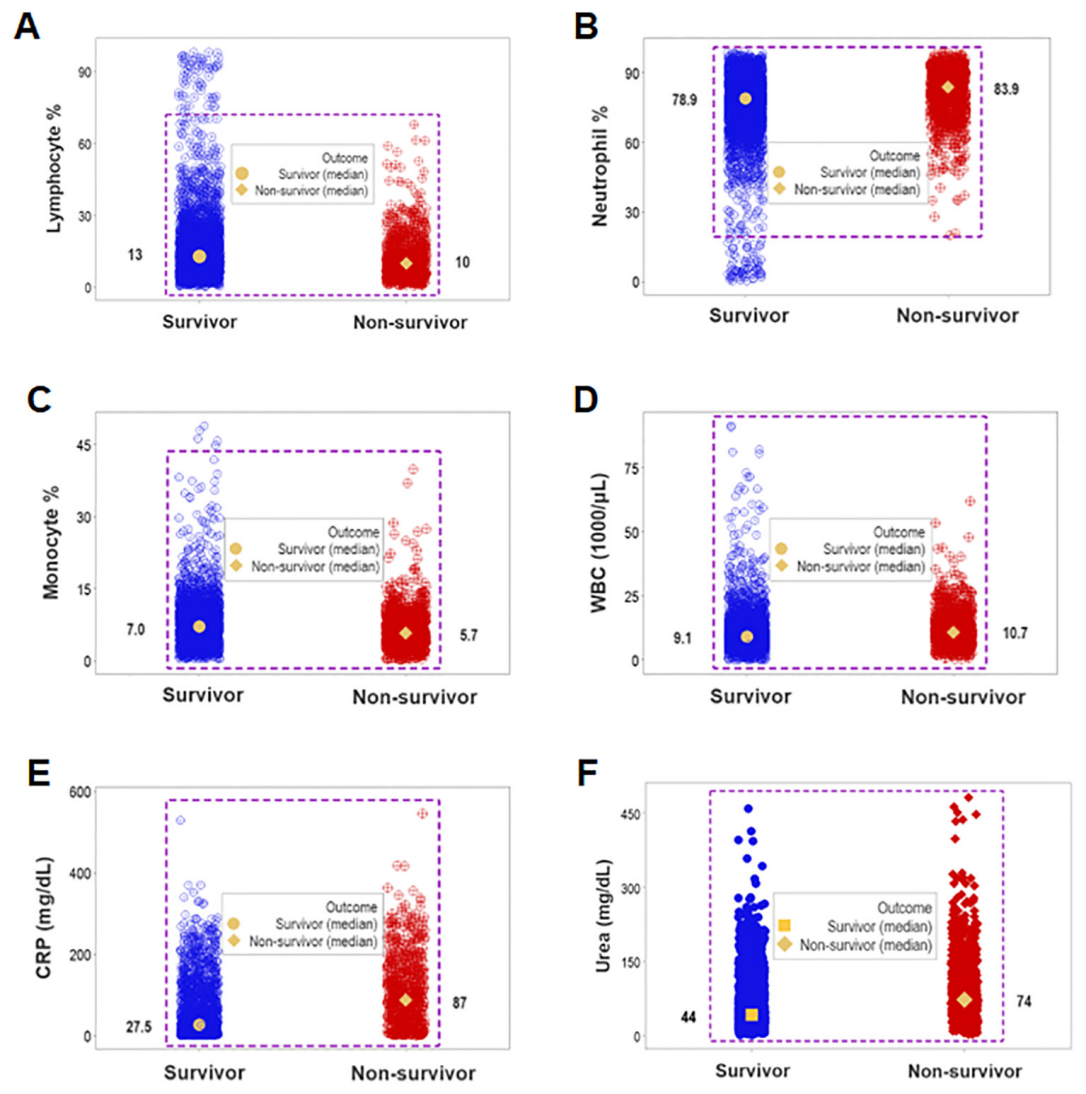
Assessment of the classic (reductionist) method (I). Overlapping cell-related and serological variables prevented outcome differentiation: unstructured data of separate cell types did not discriminate outcomes. Lack of differentiation was due to data overlapping: the leukocyte percentages or counts of most survivors were within the range reported by non-survivors (rectangles, **A–D**). Lack of differentiation also involved the blood concentration of C-Reactive Protein (CRP) and urea, which revealed overlapping distributions even when medians differed at statistically significant levels among outcomes (*p ≤*0.01, Mann-Whitney test, **E, F**).

While unstructured data (the counts or percentages of lymphocytes [L], neutrophils [N], or monocytes [M]) differed at statistically significant levels among patients who presented with opposing outcomes, *data overlapping* was observed: survivors could not be distinguished from non-survivors because the leukocyte percentages or counts of most survivors overlapped with those of most non-survivors (rectangles, **Figures 1A–D**). Even when statistically significant differences were found, data overlapping prevented medical personalized differentiations. For instance, the blood concentration of C-Reactive Protein (CRP) and the serum concentration of urea revealed overlapping intervals between survivors and non-survivors even when medians exhibited a statistically significant difference (*p ≤*0.01, Mann-Whitney test, **Figures 1E, F**).

**Figure 2:**
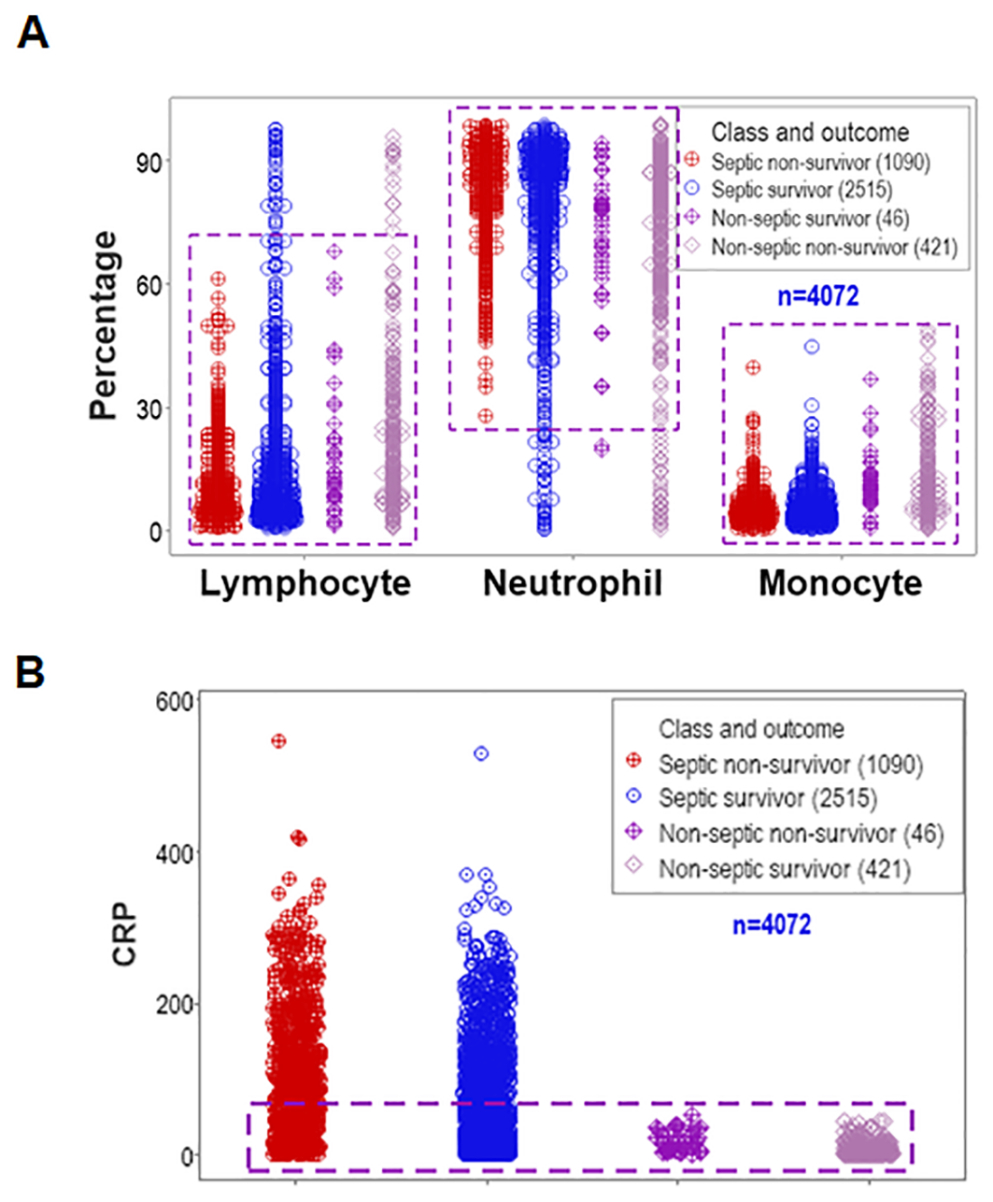
Assessment of the classic (reductionist) method (II). Discrimination did not improve when four classes of patients were considered (septic non-survivors, septic survivors, non-septic non-survivors and non-septic survivors). Overlapping data distributions are indicated by rectangles **(A, B)**.

Data overlapping remained when four classes (septic non-survivors, septic survivors, non-septic non-survivors and non-septic survivors) were considered. Despite clinical differences, cellular and non-cellular data exhibited partially similar data intervals (rectangles, **Figures 2A, B**).

### 3.3 Structured data

In contrast, when outcomes were explored with structured and temporal data (n=4087), distinct patterns differentiated two groups of patients, which markedly differed in mortality.

**Figure 3:**
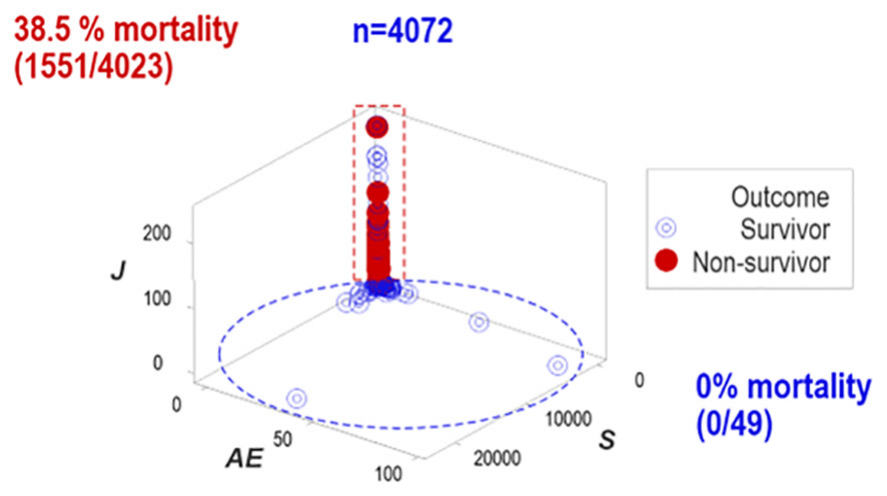
Distinct patterns of structured data. Structured data revealed non-random patterns, including data subsets perpendicular to one another. Non-overlapping data subsets differed markedly in outcomes: mortality was 0% in the horizontal subset and 38.5% in the vertical one.

One data subset revealed no mortality (horizontal oval, **Figure 3**) while the other group expressed 38.5% mortality (vertical rectangle, **Figure 3**).

**Figure 4:**
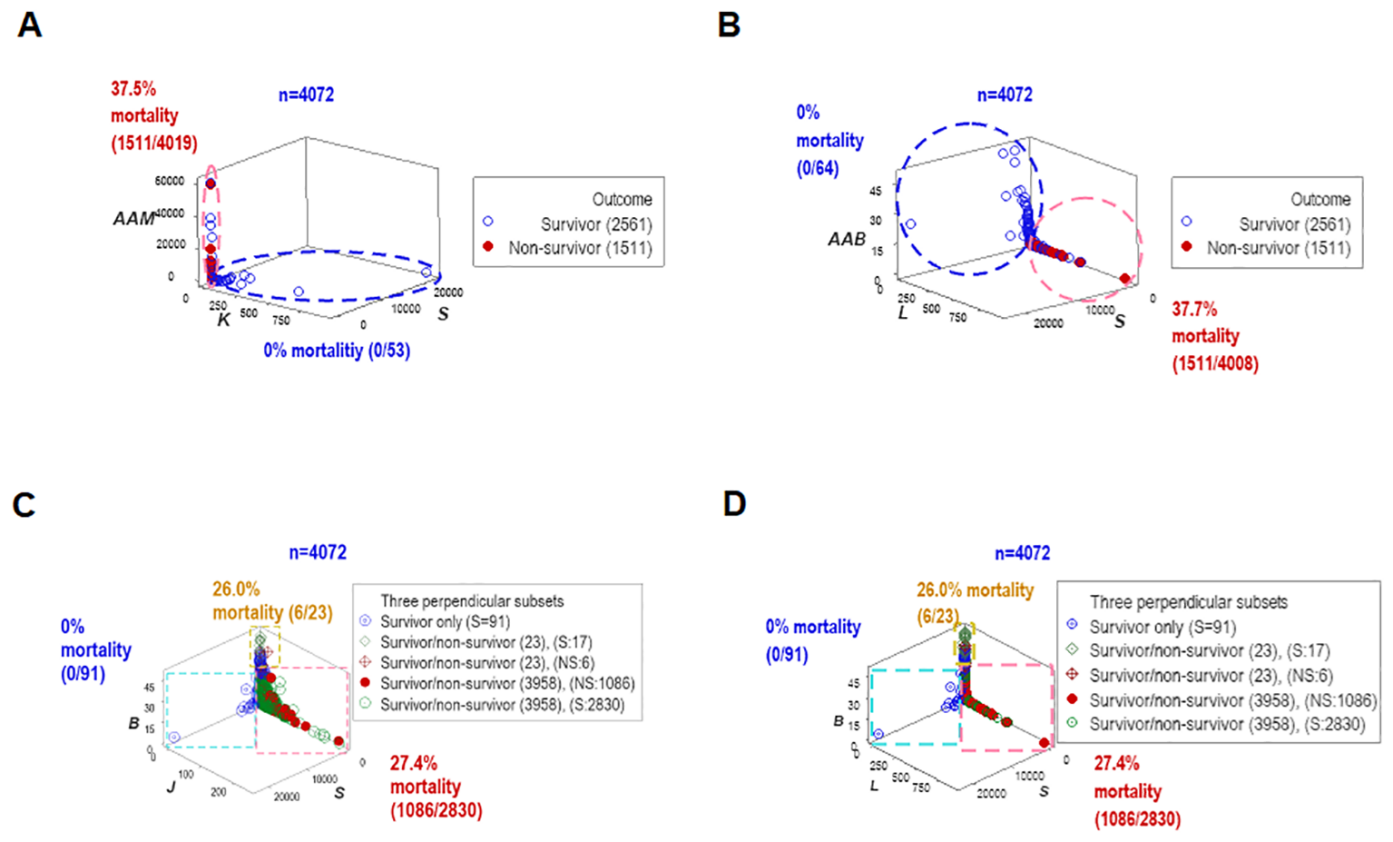
Redundant analysis: structured data reveal patterns that distinguish mortality-related groups. Four partially redundant analyses of structured data distinguished mortality-related groups. Two or more groups of patients were differentiated, who differed markedly in terms of in-hospital survival **(A–D)**. These analyses included all longitudinal observations (n=4072).

To assess repeatability, additional data structures were investigated. Redundant analyses (conducted with different data structures) distinguished two or three non-overlapping data subsets, of which one was only occupied by survivors (blue oval or rectangle, **Figures 4A–D**), and one of the remaining subsets expressed up to 37.7% mortality (red vertical oval or rectangle, **Figures 4A–D**). Septic patients represented 99.7% of the observations located within one subset (red rectangle, **Figures 4C, D**).

**Figure 5:**
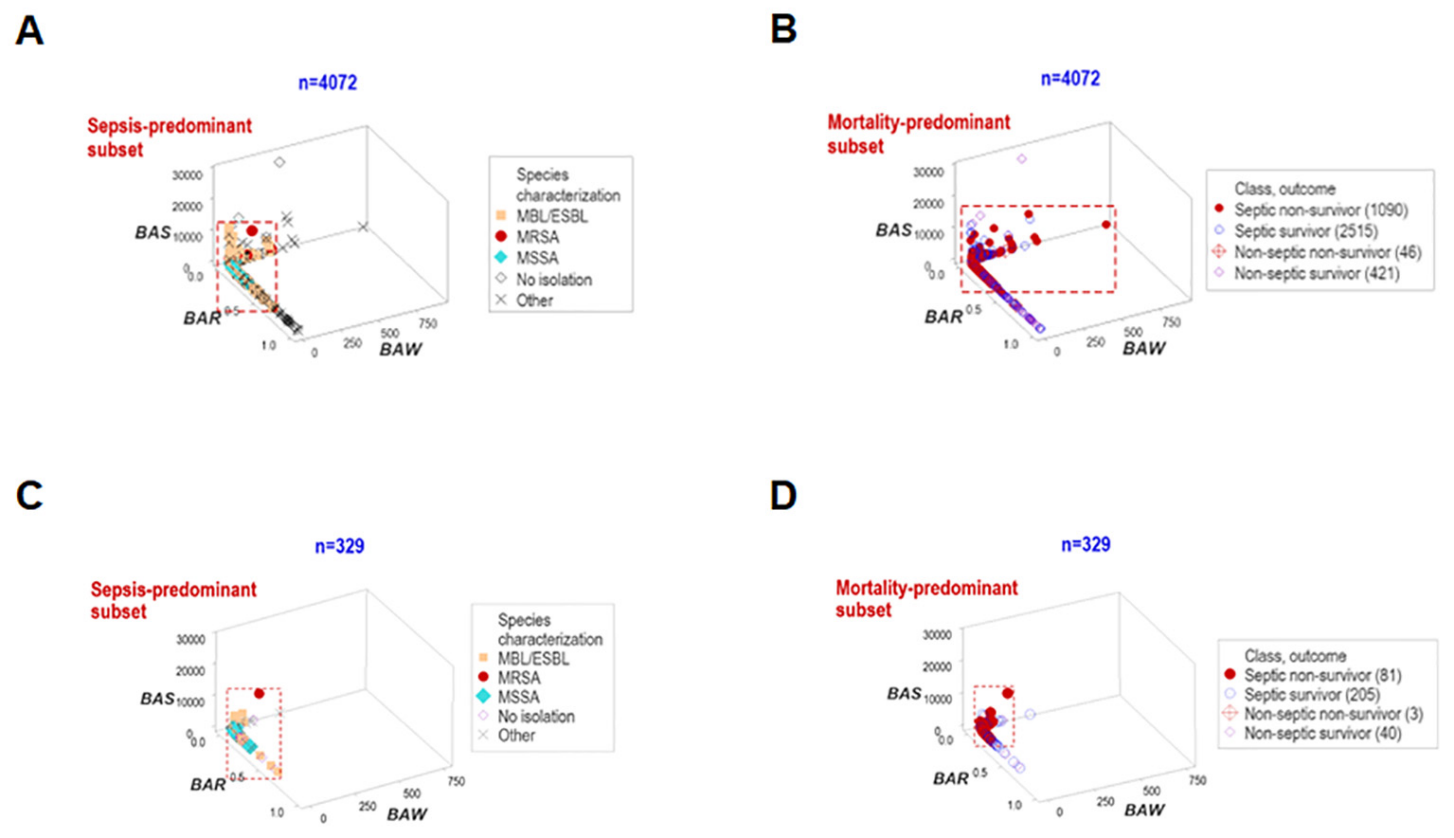
Structured data reveal patterns that distinguish bacteriological groups. Two data structures were used to analyze longitudinal bacteriological results and explore temporal findings (n=4072, **A, B**). The patient subgroups that experienced the highest mortality were infected by the ESBL, MBL, MRSA and MSSA bacterial species. Similar patterns were found when day-1 data were analyzed (n=329, **C, D**).

`Other data structures explored immunological-bacterial relationships. They involved methicillin-resistant and methicillin-sensitive *Staphylococcus aureus* (MRSA, MSSA) as well as extended-spectrum beta-lactamase (ESBL) and metallo beta-lactamase (MBL) pathogens. The ESBL/MBL subgroup was composed by *Acinetobacter baumannii*, *Escherichia coli*, and *Klebsiella pneumoniae.* These five bacterial species were associated with a small data range, which predominantly included non-survivor septic cases (red rectangle, **Figures 5A, B**). Similar patterns were also observed when hospitalization day 1 data were analyzed (**Figures 5C, D**).

### 3.4 Early prognosis

**Figure 6:**
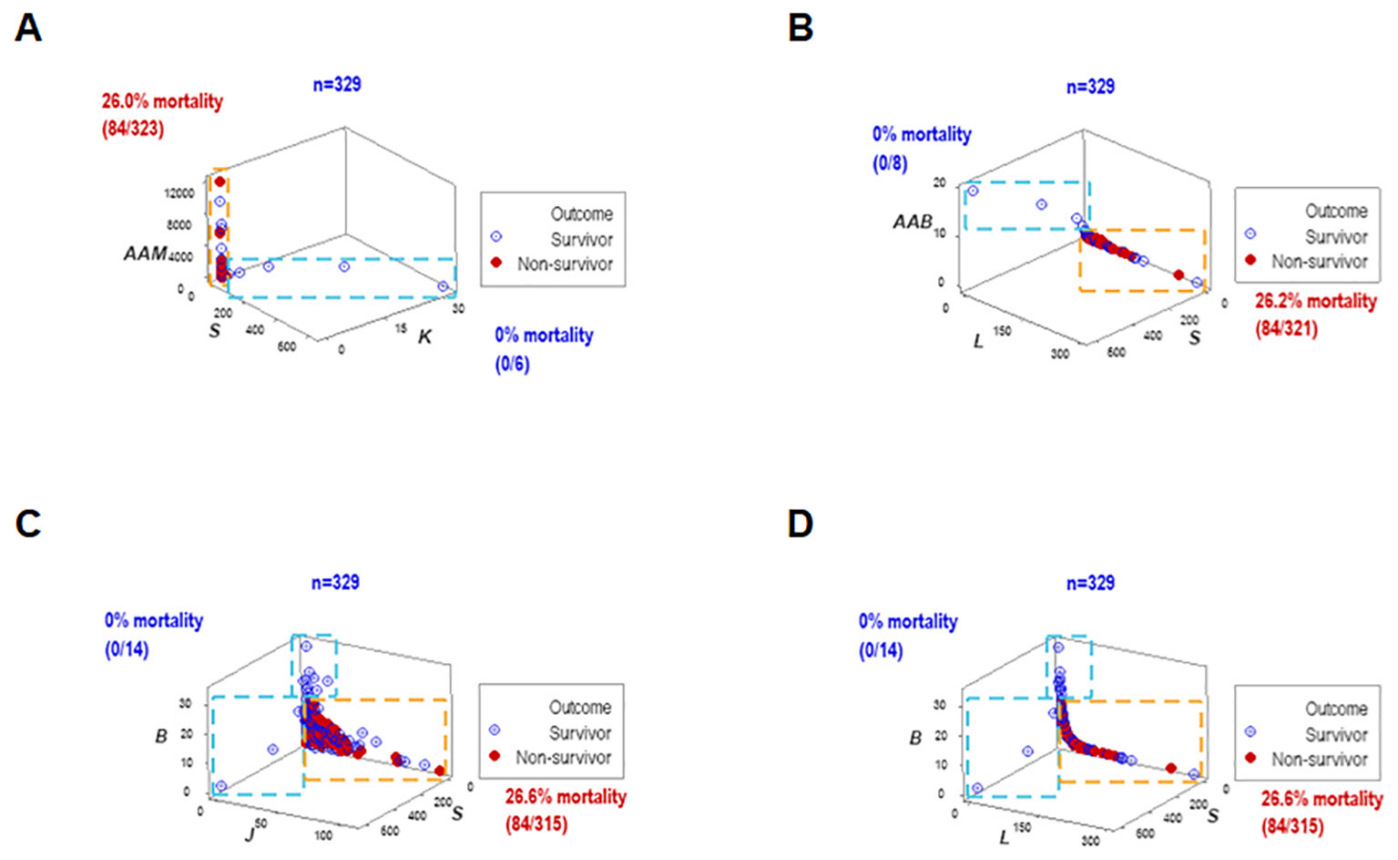
Early prognosis – I, four disease classes and bacteriological groups. When the data structures explored in **Figure 4** were re-assessed with hospitalization day 1 data, similar patterns were exhibited **(A–D)**. Therefore, data patterns that markedly differed in mortality were prognostic as early as hospitalization day 1.

To elucidate whether immuno-bacterial data patterns were prognostic, hospitalization day-1 data were assessed (n=329; **Supplementary Table 1**). Day-1 patterns corroborated earlier patterns: at least two non-overlapping data subsets were discriminated and at least one subset reported 100% survivors (**Figures 6A–D**). With one exception (the upper vertical subset where, after day 1, some non-survivors were reported (**Figures 6C, D**), all other subsets reporting 0% mortality on day 1 provided prognostic information.

### 3.5 First biological validations and prognostic impact of leukocyte-bacterial patterns

**Figure 7:**
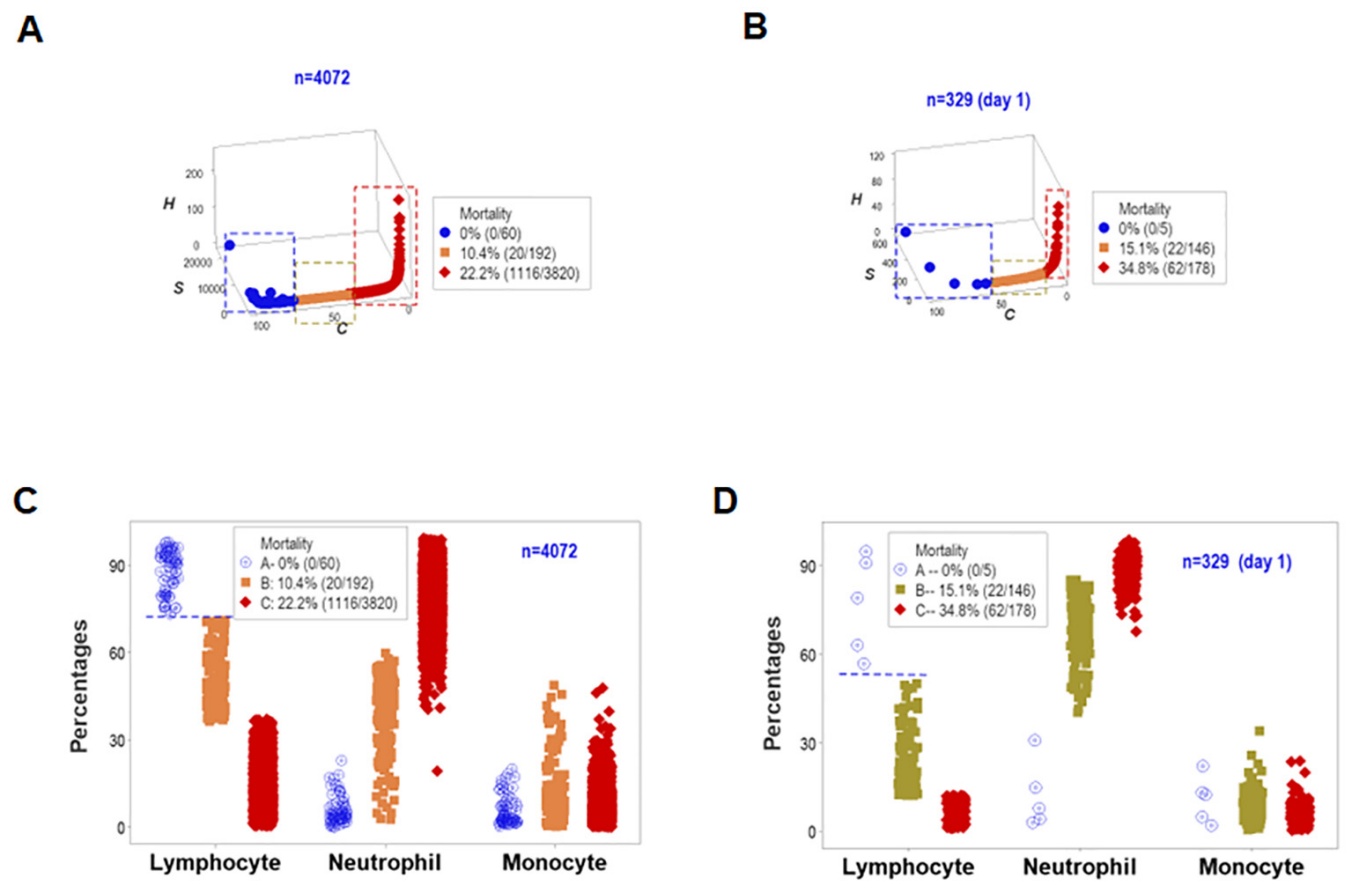
First immunological validation and first assessment of prognostic repeatability. When one data point-wide lines of observations were considered to explore triple (outcome-microbial-immunological) interactions, both the overall longitudinal dataset **(A)** and day-1 data **(B)** differentiated three data subsets that markedly differed in mortality: [1] on the left side, no mortality was reported (blue symbols, **A, B**); [2] in the middle, 10.4-15.1% mortality was found (orange symbols, **A, B**), and [3] on the right side, 22.2-34.8% fatalities were identified (red symbols, **A, B**). Such patterns were biologically validated: when lymphocyte percentages were considered, both the overall longitudinal dataset and day -1 data differentiated the 0% mortality group from all other groups without overlapping intervals (horizontal line, **C, D**).

To assess the reproducibility of the previous findings as well as their biological validity and medical (prognostic) applicability, a different set of structured data was investigated. Both the longitudinal and day-1 analyses revealed similar patterns: they showed one data point-wide lines of observations that exhibited two perpendicular data inflections in three-dimensional (3D) space (**Figures 7A, B**). Such spatial patterns were validated with a biological interpretable variable –survival. Three different levels of mortality were observed, with 100% survival on one end of the data interval (group ‘A’), between 10 and 15% mortality in the intermediate data subset (‘B”), and more than 22% fatalities in the remaining subset (‘C’, **Figures 7A, B**). A second biomedical validation was based on leukocyte data. At least the lymphocyte percentage showed non-overlapping data intervals between the subset that reported 100% survival and the remaining subsets (**Figures 7C, D**). Therefore, validity was shown (twice) as well as prognostic applications.

### 3.6 Non-binary (outcome-related, leukocyte, bacterial, temporal) data partitioning

**Figure 8:**
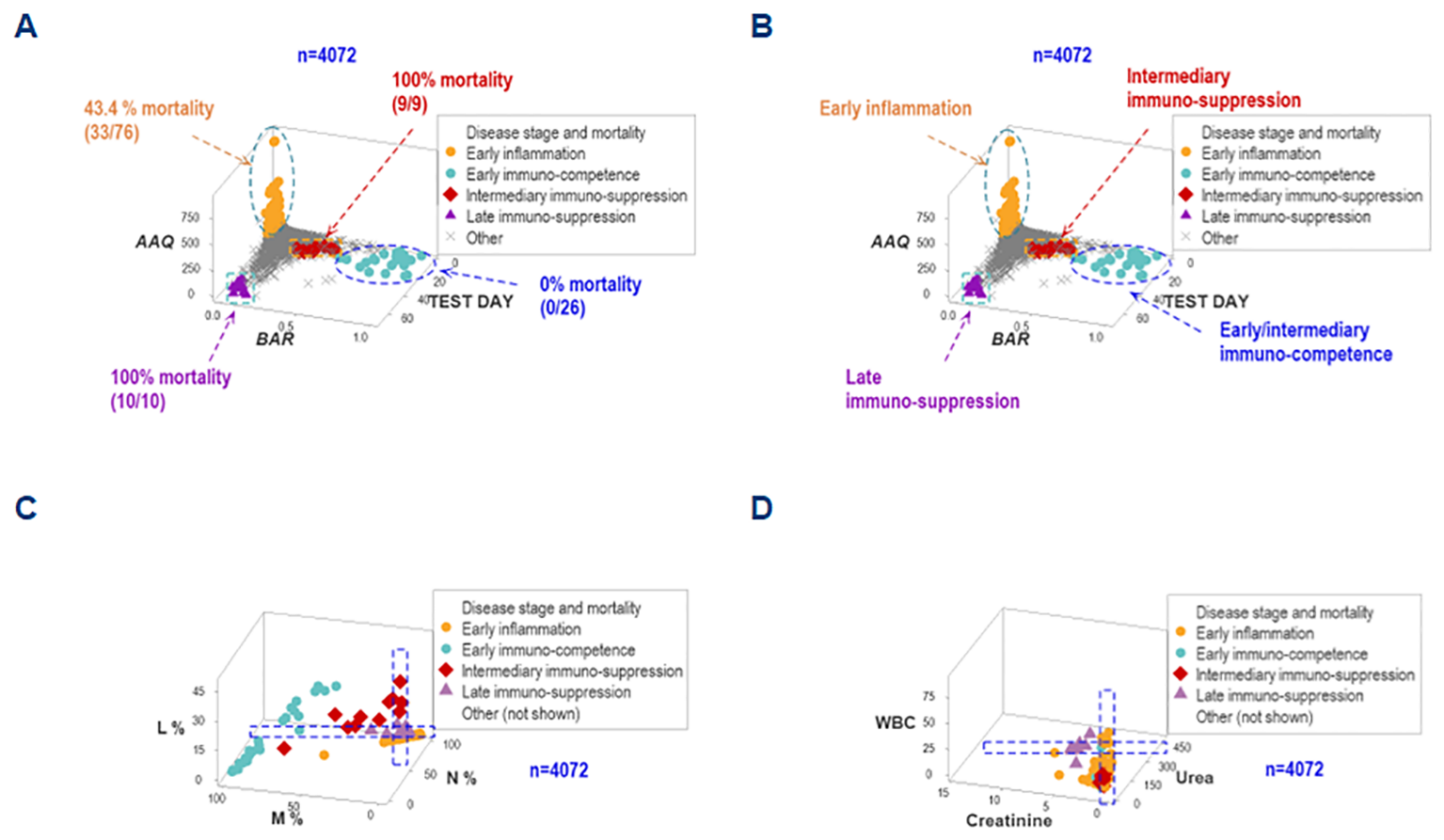
Differentiation of inflammatory stages. The simultaneous assessment of temporal-immunological-bacterial-outcome related dimensions distinguished five inflammatory stages or phases, which differed in mortality **(A, B)**. Such stages were provisionally characterized as (i) inflammation, (ii) immuno-suppression, or (iii) immunocompetence, and further subdivided into early, intermediary or late expressions. Such inflammatory stages were not identified by isolated variables: any angle or spatial perspective of the same data would reveal overlapping observations of different stages **(C, D)**.

The simultaneous assessment of outcomes and multi-dimensional inputs detected five data groups, here provisionally named (a) early inflammation, (b) early immuno-competence, (c) intermediary suppression, (d) late suppression, or (e) other. These groups differed markedly in terms of mortality: while both ‘suppression’ subsets were associated with a 100% mortality, the ‘immuno-competence’ group displayed 100% survival (**Figures 8A, B**).

The non-reductionist method did not detect such groups. Regardless of the angle or spatial perspective considered, overlapping intervals of different inflammatory stages were observed when either cellular or non-cellular variables were evaluated in isolation (**Figures 8C, D**).

### 3.7 Second statistical and biological validation of non-binary data classes

**Figure 9:**
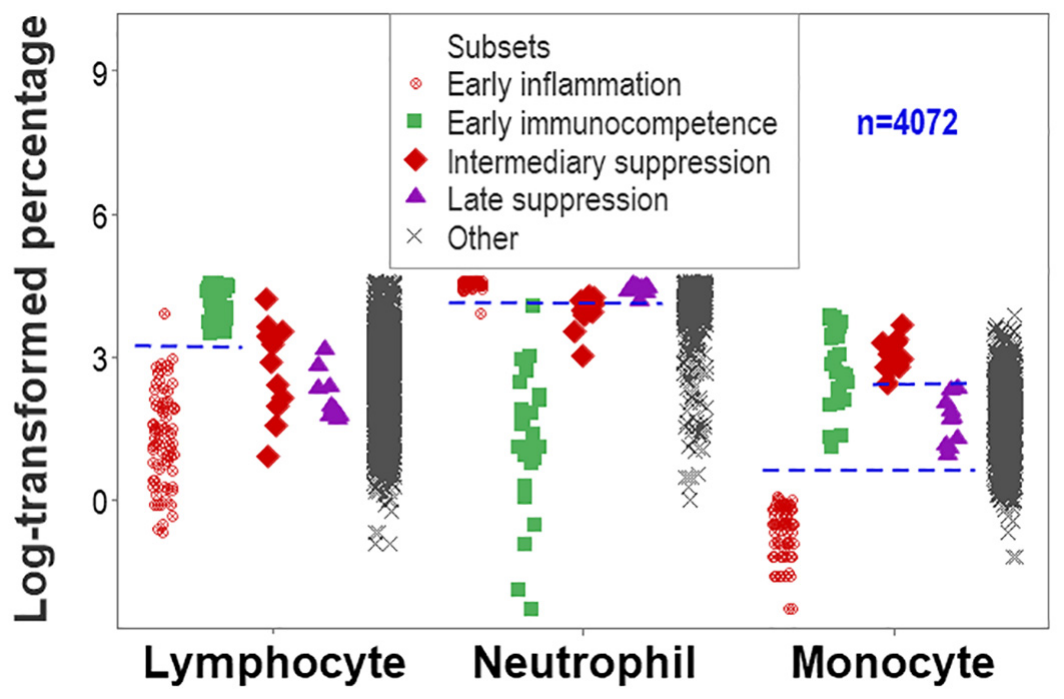
Second validation (first validation of inflammatory stages). The analysis of inflammatory phases or stages led to an increased number of non-overlapping data intervals. The lymphocyte percentage distinguished early inflammation from early immunocompetence and early immunocompetence from late suppression (also differentiated by the neutrophil percentage). The monocyte percentage differentiated (i) early inflammation from early immunocompetence, (ii) early inflammation from intermediary suppression, (iii) early inflammation from late suppression, and (iv) intermediary from late suppression.

While data overlapping was found when the method was limited to two outcomes (**Figure 1**), non-overlapping data intervals characterized the method designed to express any number of findings. For instance, non-overlapping monocyte percentage intervals distinguished the early immunocompetence from the early inflammation subsets (**Figure 9**).

Documenting that statistical significance is not equal to biomedical discrimination, the non-reductionist method was associated with more statistically significant inferences than the reductionist alternative. For example, all inflammatory phase-related analyses that reached statistical significance also exhibited overlapping data intervals when the reductionist method was used. In contrast, all comparisons based on *BAR* (a dimensionless indicator) showed both statistically significant differences and non-overlapping data intervals (**Supplementary Table 2**).

**Figure 10:**
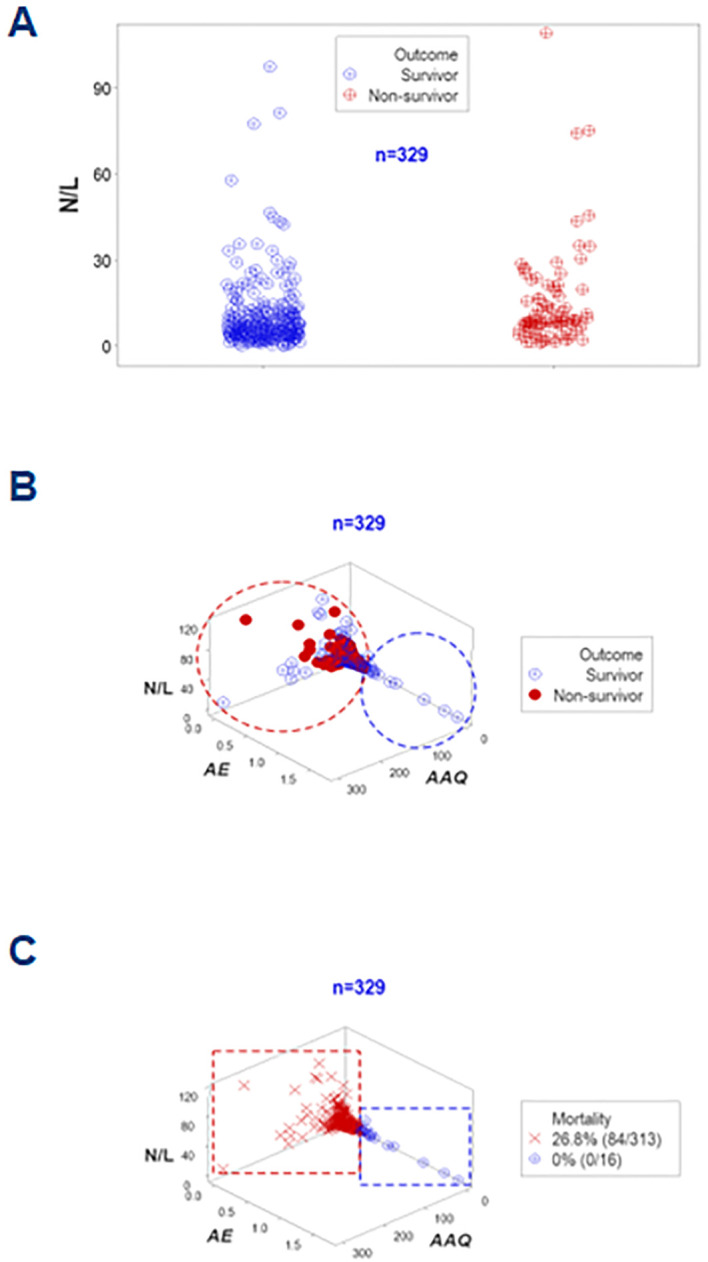
Further validation of disease stages (assessment of the N/L ratio). The neutrophil/lymphocyte or N/L ratio–a metric regarded in some publications as a marker of sepsis— was also investigated. The N/L was not informative in this study at day 1: despite statistically significantly different median N/L ratios (*p*<0.01, Mann-Whitney test), overlapping data outcomes were not differentiated by this ratio **(A)**. Yet, when combined with other indicators and explored in 3D space, two subsets that markedly differed in mortality were differentiated **(B, C)**.

An additional example of the same concept involved the neutrophil/lymphocyte (N/L) ratio. Even when the median N/L differed significantly between survivors and non-survivors (6.28 vs. 8.78, respectively, *p*<0.01, Mann-Whitney test, **Figure 10A**), data overlapping inhibited clinicians to separate survivors from non-survivors. However, when integrated with other metrics, the N/L distinguished subsets that differed in mortality (**Figures 10B, C**).

The analysis of kurtosis (a measure of peakedness) added usable information. While septic cases showed kurtosis when leukocytes were measured in isolation, when inflammatory stages were considered, kurtosis was only reported in week 1 (**Supplementary Table 3**).

### 3.8 A third biological validation and additional possible clinical applications

**Figure 11:**
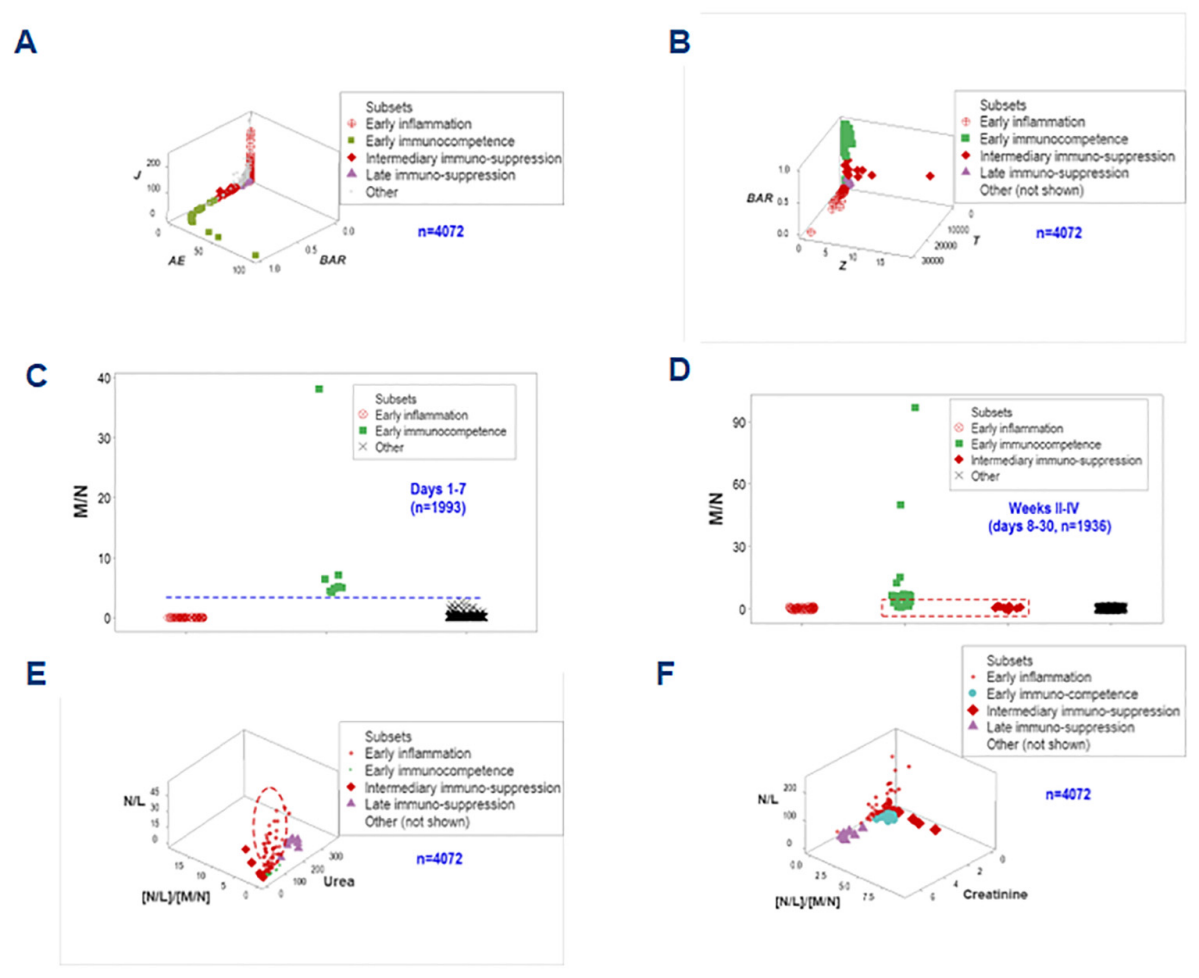
Third biological validation and additional clinical applications. Two one data point-wide lines of observations helped distinguishing the inflammatory phase-related data groups **(A, B)**. When time was considered and biologically interpretable indicators were explored, it was shown that no immunosuppression took place in the first hospitalization week, when immunocompetence was characterized by increased monocyte/neutrophil ratios **(C)**. Early immunocompetence was short-lived: it was not documented in weeks 2-4 –when immunosuppression was first detected **(D)**. N/L, together with the [N/L]/[M/N] ratios and the serum concentration of urea or creatinine helped differentiating the intermediary (earlier) from the late suppression: the former was associated with increases in both N/L and [N/L]/[M/N] ratios (i.e., a decreased monocyte-related function) while the latter was characterized by renal dysfunction (increased urea or creatinine concentrations, **E, F**).

To enhance discrimination, time and two more data structures were examined. They showed data inflections that separated two subsets (immunocompetence and early inflammation) from the remaining groups (**Figures 11A, B**). While immuno-suppression was not detected in hospitalization week 1, immuno-competence was only observed in the first week (**Figures 11C, D**).

Biomedically interpretable variables validated the inflammatory phases identified above (1): early immuno-competence revealed urea concentrations and N/L ratio values approaching zero; (2) the ‘intermediary’ suppression (observed at or after the early inflammation but before the late suppression) expressed both increased N/L and increased [N/L]/[M/N] ratio values, i.e., a monocyte-mediated immuno-suppression; (3) the late suppression was characterized by increased serum concentrations of urea and creatinine (renal dysfunction); and (4) the early inflammation displayed high N/L values while the [N/L/[M/M] ratios approached zero and this group was orthogonal to both types of suppression (**Figures 11E, F**). These spatial data patterns possessed clinical applications: they could distinguish the intermediary from the late suppression.

### 3.9 Personalized assessments

**Figure 12:**
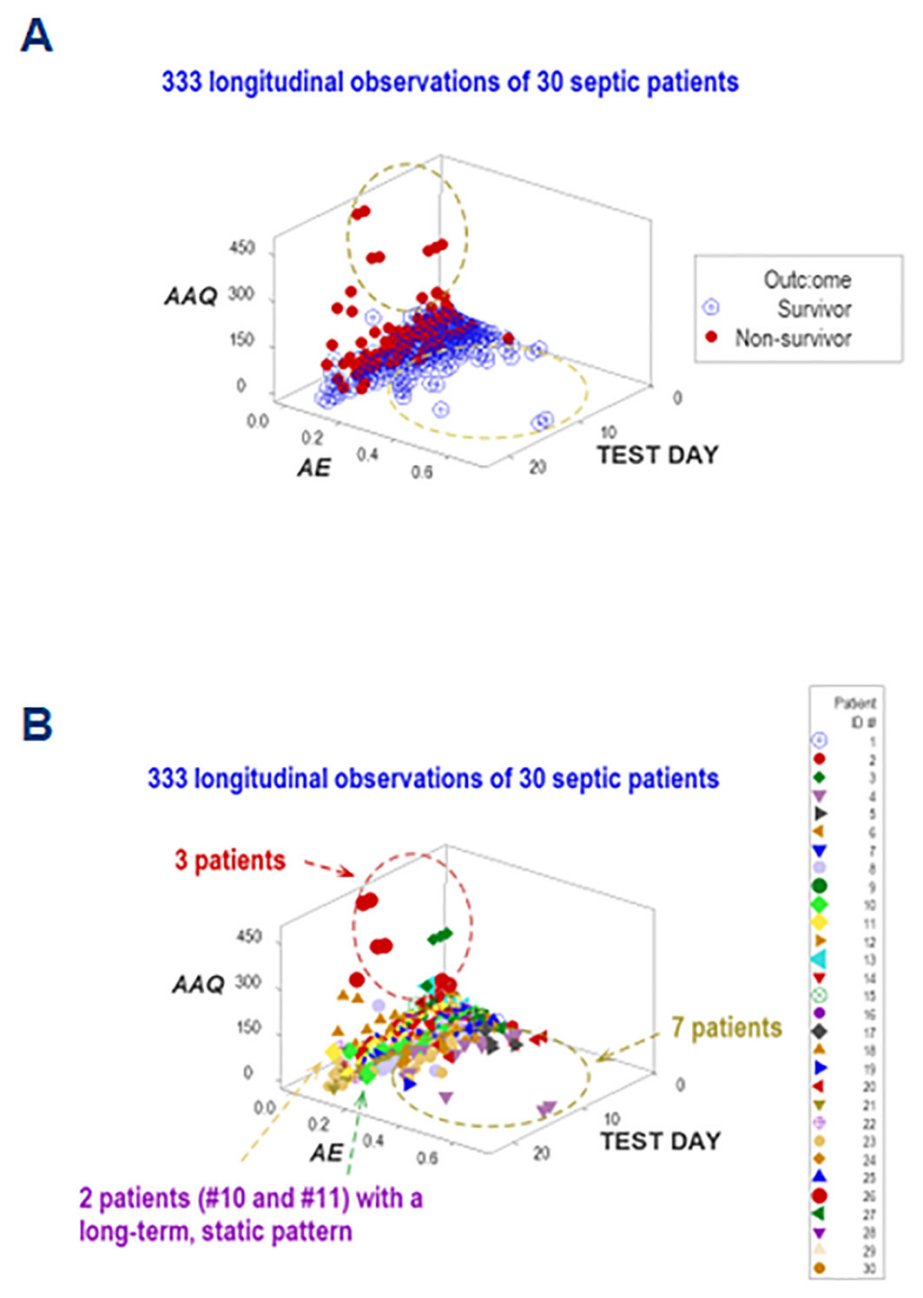
Personalized assessments. Additional information emerged when five perspectives were simultaneously investigated (*immune-microbial-clinical* [mortality-related]-*temporal-personalized* interactions). When a subset of observations was evaluated (which included 30 patients who contributed 333 longitudinal observations), three data subsets were differentiated, which are consistent with either inflammation, immuno-competence, or immune-suppression **(A)**. The last classification was supported by the outcome and the long-term lack of variability exhibited by two patients (#10 and 11, **(B)**.

Five dimensions were simultaneously assessed when, in addition to disease stage, outcomes, and immuno-bacterial profiles, patients were individually explored. Given the high number of patients included in this study, the personalized analysis only considered 30 septic patients who contributed 330 longitudinal observations. They revealed patterns characterized by either mortality or survival (**Figure 12A**). When the analysis was focused on specific patients, three profiles (inflammation, immunocompetence, and immunosuppression) emerged (**Figure 12B**). Two patients (#10 and #11) exhibited unaltered profiles over several consecutive weeks, suggesting immunosuppression (**Figure 12B**). Hence, personalized and temporal assessments may provide additional information, including patterns that resemble long-term immuno-paralysis.

**Figure 13:**
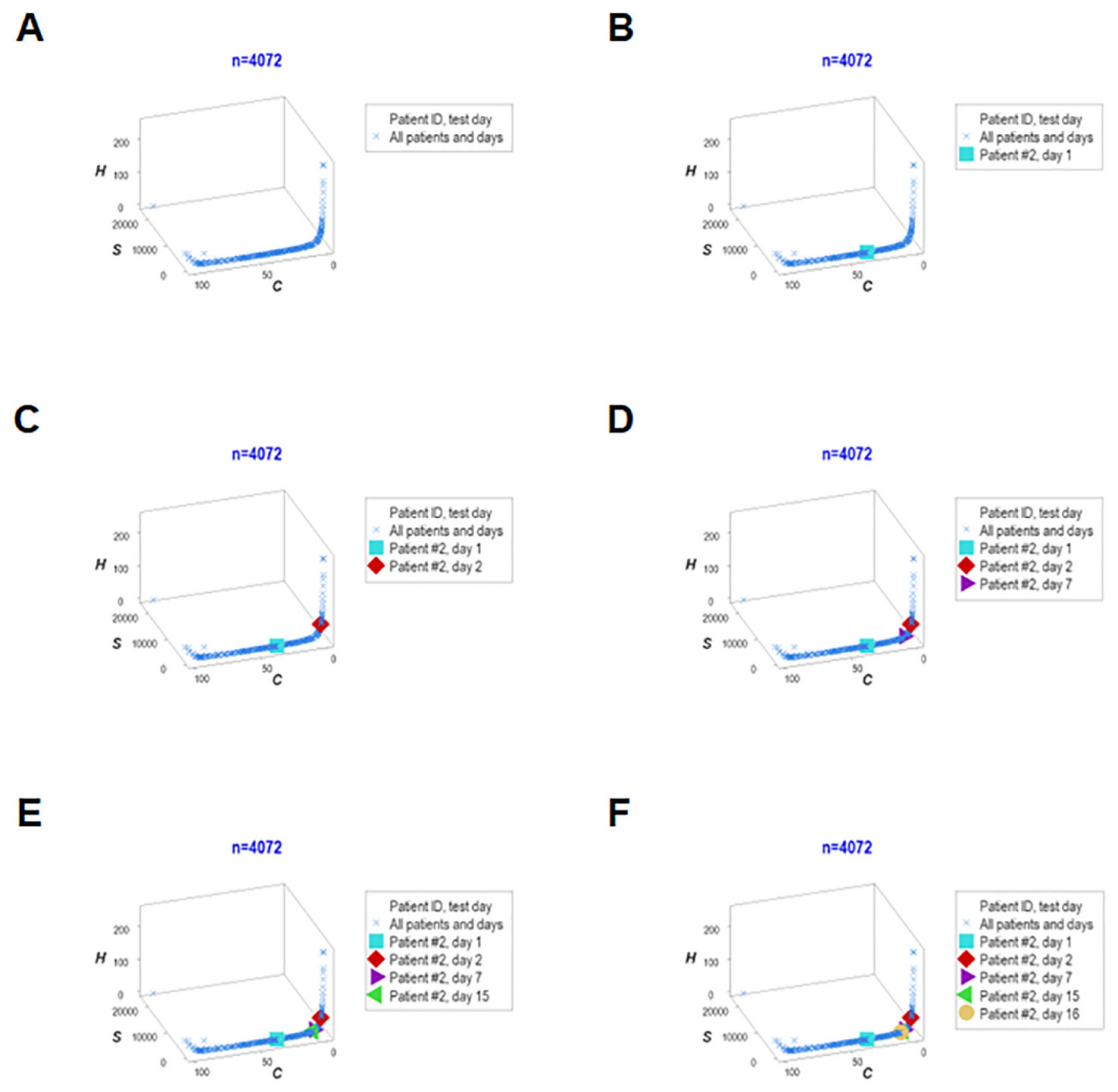
Real-time, personalized and population assessments. The last assessment of this study considered six dimensions. In addition to the previously reported dimensions, the population-related information was used as a reference. This approach detected changes in directionality even in small units of time, i.e., in real time. When disease trajectory was considered, the simultaneous assessment of *immune-microbial-clinical* [mortality-related]-*temporal*-*personalized* and *population*-related interactions provided prognostic information. Detection was not based on numerical (quantitative) but qualitative information: changes in the directionality of the data (arrows) were considered. This personalized and population-based detection system can express, in real time, personalized changes in directionality (arrows that point at different directions) and relate such movements to the overall (population-level) data. This system is illustrated with data pertaining to one specific patient (‘patient #2’), who was tested five times –at days 1, 3, 5, 9 and 18 **(A–F)**. It is shown that, in every test, ‘patient #2’ remained within the ‘right’ end of the data distribution (the data segment associated with the highest mortality, as shown in **Figures 7A, B**).

Adding population-related data to the dimensions previously evaluated, the last analysis explored six interactions (**Figures 13A–F**). One patient (‘patient 2’) showed, on five occasions, that every observation fell within the data region associated with the highest mortality. Thus, this personalized/population pattern predicted non-survival –an outcome observed in ‘patient 2.’

## 4 Discussion

### 4.1 Overview

This study investigated (a) the reductionist method (**Figures 1**, **2**); (b) the validity of the non-reductionist alternative (**Figures 3**–**7**); and (c) six (personalized-, population- temporal-, bacterial-, immunological-, and outcome-related) dimensions (**Figures 7**–**13**). Validated three times with biologically interpretable variables associated with statistical significance, findings showed that more information can be extracted from the same data when many interactions and dimensions are tested. Theoretical considerations and possible applications are discussed below.

### 4.2 Theoretical considerations

#### 4.2.1 Validation

Construct, internal, external, and statistical types of validity were explored (50).

Construct validity answers this question: *are we testing what we need to test?* A valid construct does not measure anything conveniently measured (which may be irrelevant) but relevant concept(s). To demonstrate construct validity, the non-reductionist method should inform more, better and/or earlier than the alternative. The non-reductionist approach revealed construct validity in all tested dimensions.

To demonstrate internal validity, a novel method should be robust (51). This study used numerous and (at least partially) different data structures that induced similar inferences.

To document external validity, a new method should apply to different populations, sites and/or time points (52). Because day-1 data prognosticated, external validity was supported.

Additional evidence of external validity is the fact that this study corroborated earlier studies on sepsis, which utilized the same methodology (45).

Findings showed that statistical significance is not always informative (53). To indicate statistical and biomedical validity, non-overlapping data distributions should also be documented (54). The non-reductionist method increased the number of assessments that revealed both non-overlapping data and statistically significant differences (**Supplementary Table 2**).

The non-reductionist approach also diminished the frequency of kurtosis (**Supplementary Table 2**). Hence, combining this method with statistical analyses could improve both.

#### 4.2.2 Reductionist vs. non-reductionist predictions

While the reductionist method failed to separate outcomes (**Figures 1**, **2**), the non-reductionist alternative discriminated outcomes and provided new information (**Figures 3**–**13**).

Multi-dimensional analyses enhanced discrimination even though the same data did not induce inferences when analyzed with the reductionist approach (**Figures 8C, D**, **9**).

While reductionist approaches tend to make assumptions before data collection, non-reductionist alternatives may be assumption-free, postulating hypotheses only after the data are collected (31). This difference leads to a major methodological consequence: the possibility of detecting biologically different conditions regardless of the shape shown by the data.

One possible reason why non-reductionist methods inform more and/or better is because they can capture dynamic interactions occurring between blood cells and the surrounding endothelium (55, 56). Reductionist methods do not inform on such a critical relationship and do not consider that temporal relationships may be asynchronous and complex. While reductionist methods emphasize *entities* (e.g., one molecule or one cell type), non-reductionist alternatives focus on *relationships* among entities –not entities themselves (57, 58).

### 4.3 Potential applications

#### 4.3.1 Visualizations of critical biological functions

It is suggested that hematological data could be routinely expressed as 3D visualizations (59, 60). They can reveal patterns two-dimensional (2D or tabular) data cannot express.

#### 4.3.2 Early patient partitioning

Findings distinguished at least three groups of patients as early as hospitalization day 1. Such a finding discriminated earlier than alternative tests (41).

#### 4.3.3 Differentiation of inflammatory stages, including two types of suppression

To the best of our knowledge, this is the first study that differentiated two varieties of sepsis-related *suppression*. While several tests have explored immunosuppression and differentiated inflammatory from non-inflammatory (kidney-mediated) suppression, earlier tests did not distinguish septic from non-septic patients with non-overlapping data intervals (61–63).

The method under study examined data abundantly available (the CBC), which were rapidly analyzed with a software package. Because the one here used is still under evaluation, readers interested in using this methodology may request assistance from the authors.

New sepsis-related methods may consider the discrimination documented in **Figures 8A–D** as well as the ability of the non-reductionist approach to analyze, later, cell surface-related variables (64, 65). Furthermore, responses characterized by immuno-paralysis may consider the personalized and temporal approach described in **Figures 12A, B** (66).

#### 4.3.4 Evaluation of previously reported and new indicators

Findings helped rectify ratio-based inferences. While increased neutrophil/lymphocyte (N/L) ratios have been reported in sepsis (67, 68), earlier studies did not focus on longitudinal and personalized data. Because this study showed N/L ratio increases in some but not all early observations (**Figures 10A–C**), it is suggested that, to avoid erroneous generalizations, ratio-related inferences should be grounded on personalized data, not populations.

Some AI-based studies on sepsis have reported poor external validity (69–73). Given the external validity of this method, future AI studies could integrate both approaches.

#### 4.3.5 Personalized immuno-modulatory therapies

Immuno-modulation has been proposed to treat sepsis (61, 74) Such therapies require personalized strategies. While earlier methods have emphasized pathogens −not individual patients (75) −, this study pursued both approaches.

### 4.4 Caveats and future studies

Two limitations are identified: (i) many unclassified observations (the ‘other’ group), and (ii) relevant dimensions not yet tested. Future studies may address these caveats investigating (a) antibiograms, i.e., microbial-antibiotic interactions (76); and (b) the dynamics of the ‘other’ group, which may require personalized inquiries on sub-cellular levels (65).

## 5 Summary and conclusions

Findings supported the central concept of the new method: a new description of immuno-suppression. Unlike earlier approaches (which defined immuno-suppression before data were collected, e.g., ‘*lymphopenia indicates immunosuppression*’), the method here explored was data-driven, visual, and validated with biomedically relevant, internally homogeneous data subsets, such as those reporting 100% survival. It can (i) detect suppression even when lymphopenia is not observed (77), (ii) differentiate inflammatory from non-inflammatory suppression (78), and (iii) offer information that may support personalized therapies (79). In sepsis, such a method may also promote, prevent or evaluate: [1] *early (day-1) patient partitioning*; [2] *confounding* (no data overlapping); [3] kurtosis-associated errors; [4] alone or together with other techniques*, detection of function(s)*, e.g., immuno-competence testing; and [5] *personalized immuno-modulation.*

## Data Availability

The datasets presented in this study can be found in online repositories. The names of the repository/repositories and accession number(s) can be found in the article/**Supplementary Material**.

## In memoriam

This paper is dedicated to the memory of Theodora Pappa, who passed away on 23/12/2023.
