# Supplementary material for: Personalized, disease-stage specific, rapid identification of immunosuppression in sepsis
Source: Front Immunol. 2024 Oct 29;15:1430972. doi: 10.3389/fimmu.2024.1430972 (PMC11558526; doi:10.3389/fimmu.2024.1430972)
Supplement: Supplementary file 1 [file Table1.docx]

Table 1. Day-1 leukocyte, bacteriological and mortality-related personalized information (286 septic- and 43 non-septic-related [329] observations).

| **ID^A^** | **Outcome^B^** | **Class^C^** | **WBC^D^** | **N%^E^** | **M%^F^** | **L%^G^** | **AE^H^** | **AAQ^I^** | **BAR^J^** | **CRP^K^** | **Bacter^L^** | **Day-1 pp.^M^** |
| --- | --- | --- | --- | --- | --- | --- | --- | --- | --- | --- | --- | --- |
| 1 | 0 | 1 | 4.9 | 48.9 | 9.1 | 42.1 | 0.19 | 6.4 | 0.16 | 30.7 | Other | High (26.8%) |
| 2 | 1 | 1 | 4.2 | 51.8 | 12.6 | 35.7 | 0.24 | 5.1 | 0.20 | 4.9 | MBL/ESBL | High (26.8%) |
| 3 | 1 | 1 | 40.7 | 98.7 | 0.4 | 0.9 | 0.00 | 246.5 | 0.00 | 192.5 | MRSA | High (26.8%) |
| 4 | 0 | 1 | 5.0 | 46.8 | 11.1 | 42.1 | 0.24 | 5.2 | 0.19 | 4.4 | MBL/ESBL | High (26.8%) |
| 5 | 0 | 1 | 13.6 | 73.2 | 8.9 | 17.9 | 0.12 | 9.3 | 0.11 | 98.6 | Other | High (26.8%) |
| 6 | 0 | 1 | 2.2 | 46.9 | 4.7 | 48.4 | 0.10 | 11.0 | 0.09 | 7.6 | Other | High (26.8%) |
| 7 | 0 | 1 | 22.9 | 85.7 | 7.1 | 7.3 | 0.08 | 13.1 | 0.08 | 106.7 | MRSA | High (26.8%) |
| 8 | 0 | 1 | 9.7 | 80.1 | 5.5 | 14.5 | 0.07 | 15.7 | 0.06 | * | Other | High (26.8%) |
| 9 | 0 | 1 | 16.9 | 80.7 | 10.6 | 8.7 | 0.13 | 8.6 | 0.12 | * | MBL/ESBL | High (26.8%) |
| 10 | 1 | 1 | 11.6 | 79.3 | 8.9 | 11.9 | 0.11 | 9.9 | 0.10 | 181.0 | Other | High (26.8%) |
| 11 | 1 | 1 | 14.8 | 91.8 | 3.8 | 4.4 | 0.04 | 25.1 | 0.04 | 69.4 | MBL/ESBL | High (26.8%) |
| 12 | 0 | 1 | 2.4 | 48.7 | 7.5 | 43.8 | 0.15 | 7.5 | 0.13 | 30.2 | Other | High (26.8%) |
| 13 | 1 | 1 | 8.3 | 89.1 | 5.4 | 5.5 | 0.06 | 17.5 | 0.06 | 97.5 | Other | High (26.8%) |
| 14 | 1 | 1 | 8.5 | 90.0 | 2.0 | 8.0 | 0.02 | 46.0 | 0.02 | 150.7 | MBL/ESBL | High (26.8%) |
| 15 | 0 | 1 | 32.4 | 86.6 | 2.3 | 11.1 | 0.03 | 38.0 | 0.03 | 122.4 | MSSA | High (26.8%) |
| 16 | 1 | 1 | 6.1 | 66.2 | 1.8 | 32.0 | 0.03 | 37.8 | 0.03 | 14.2 | MBL/ESBL | High (26.8%) |
| 17 | 0 | 1 | 10.4 | 90.4 | 2.8 | 6.8 | 0.03 | 33.1 | 0.03 | 145.0 | MBL/ESBL | High (26.8%) |
| 18 | 1 | 1 | 21.3 | 90.7 | 1.7 | 7.6 | 0.02 | 54.2 | 0.02 | 100.9 | MBL/ESBL | High (26.8%) |
| 19 | 0 | 1 | 15.6 | 89.9 | 6.5 | 3.6 | 0.07 | 14.8 | 0.07 | 228.1 | Other | High (26.8%) |
| 20 | 0 | 1 | 6.7 | 56.8 | 20.4 | 22.8 | 0.36 | 3.8 | 0.26 | 123.8 | Other | Low (0%) |
| 21 | 0 | 1 | 6.5 | 85.7 | 6.2 | 8.1 | 0.07 | 14.8 | 0.07 | 88.2 | MBL/ESBL | High (26.8%) |
| 22 | 0 | 1 | 12.4 | 80.1 | 6.2 | 13.7 | 0.08 | 13.9 | 0.07 | 35.4 | Other | High (26.8%) |
| 23 | 0 | 1 | 9.0 | 75.6 | 10.0 | 14.3 | 0.13 | 8.5 | 0.12 | 53.0 | Other | High (26.8%) |
| 24 | 0 | 1 | 6.0 | 79.9 | 8.4 | 11.8 | 0.10 | 10.6 | 0.09 | 7.3 | Other | High (26.8%) |
| 25 | 0 | 1 | 22.0 | 78.8 | 14.0 | 7.2 | 0.18 | 6.6 | 0.15 | 19.3 | Other | High (26.8%) |
| 26 | 1 | 1 | 10.5 | 92.2 | 1.2 | 6.6 | 0.01 | 77.8 | 0.01 | 3.4 | Other | High (26.8%) |
| 27 | 0 | 1 | 13.4 | 70.3 | 9.9 | 19.8 | 0.14 | 8.1 | 0.12 | 3.0 | Other | High (26.8%) |
| 28 | 0 | 1 | 13.3 | 83.2 | 4.6 | 12.3 | 0.06 | 19.1 | 0.05 | 130.5 | MRSA | High (26.8%) |
| 29 | 0 | 1 | 9.5 | 88.2 | 4.4 | 7.4 | 0.05 | 21.0 | 0.05 | 124.5 | Other | High (26.8%) |
| 30 | 0 | 1 | 12.7 | 73.3 | 8.5 | 18.2 | 0.12 | 9.6 | 0.10 | 179.2 | MSSA | High (26.8%) |
| 31 | 0 | 1 | 4.6 | 79.2 | 9.4 | 11.4 | 0.12 | 9.5 | 0.11 | 2.4 | MBL/ESBL | High (26.8%) |
| 32 | 0 | 1 | 16.8 | 85.2 | 7.3 | 7.4 | 0.09 | 12.6 | 0.08 | 251.7 | MRSA | High (26.8%) |
| 33 | 0 | 1 | 11.8 | 84.2 | 4.0 | 11.8 | 0.05 | 22.2 | 0.05 | 231.2 | MBL/ESBL | High (26.8%) |
| 34 | 0 | 1 | 8.7 | 78.3 | 7.4 | 14.3 | 0.09 | 11.5 | 0.09 | 145.5 | MBL/ESBL | High (26.8%) |
| 35 | 0 | 1 | 9.1 | 51.6 | 19.8 | 28.5 | 0.38 | 3.6 | 0.28 | 46.4 | MSSA | Low (0%) |
| 36 | 0 | 1 | 7.5 | 74.6 | 11.3 | 14.1 | 0.15 | 7.6 | 0.13 | 10.7 | MSSA | High (26.8%) |
| 37 | 1 | 1 | 20.5 | 70.0 | 11.2 | 18.8 | 0.16 | 7.2 | 0.14 | 269.9 | MSSA | High (26.8%) |
| 38 | 0 | 1 | 9.8 | 97.5 | 1.3 | 1.2 | 0.01 | 75.9 | 0.01 | 24.4 | MBL/ESBL | High (26.8%) |
| 39 | 0 | 1 | 9.7 | 83.7 | 7.5 | 8.8 | 0.09 | 12.1 | 0.08 | 2.3 | Other | High (26.8%) |
| 40 | 1 | 1 | 5.1 | 97.9 | 0.8 | 1.3 | 0.01 | 123.3 | 0.01 | 39.0 | MBL/ESBL | High (26.8%) |
| 41 | 1 | 1 | 4.9 | 90.6 | 3.9 | 5.4 | 0.04 | 24.1 | 0.04 | 124.8 | MBL/ESBL | High (26.8%) |
| 42 | 1 | 1 | 14.1 | 83.9 | 6.3 | 9.8 | 0.07 | 14.4 | 0.07 | 116.8 | MBL/ESBL | High (26.8%) |
| 43 | 1 | 1 | 22.3 | 84.9 | 7.2 | 7.9 | 0.08 | 12.8 | 0.08 | 139.9 | MBL/ESBL | High (26.8%) |
| 44 | 0 | 1 | 6.4 | 48.5 | 22.9 | 28.6 | 0.47 | 3.1 | 0.32 | * | Other | Low (0%) |
| 45 | 1 | 1 | 10.0 | 82.6 | 7.4 | 10.1 | 0.09 | 12.2 | 0.08 | 277.6 | MSSA | High (26.8%) |
| 46 | 0 | 1 | 10.5 | 68.4 | 10.6 | 21.0 | 0.15 | 7.5 | 0.13 | 206.8 | MBL/ESBL | High (26.8%) |
| 47 | 0 | 1 | 22.4 | 88.4 | 3.2 | 8.4 | 0.04 | 28.6 | 0.03 | 204.6 | MBL/ESBL | High (26.8%) |
| 48 | 1 | 1 | 23.3 | 83.0 | 7.8 | 9.2 | 0.09 | 11.6 | 0.09 | 140.6 | MBL/ESBL | High (26.8%) |
| 49 | 1 | 1 | 26.3 | 81.2 | 9.5 | 9.4 | 0.12 | 9.6 | 0.10 | * | Other | High (26.8%) |
| 50 | 0 | 1 | 8.4 | 64.5 | 8.4 | 27.1 | 0.13 | 8.7 | 0.11 | 85.5 | MBL/ESBL | High (26.8%) |

Table 1 (cont’d)

| **ID^A^** | **Outcome^B^** | **Class^C^** | **WBC^D^** | **N%^E^** | **M%^F^** | **L%^G^** | **AE^H^** | **AAQ ^I^** | **BAR^J^** | **CRP^K^** | **Bacter^L^** | **Day-1 pp.^M^** |
| --- | --- | --- | --- | --- | --- | --- | --- | --- | --- | --- | --- | --- |
| 51 | 1 | 1 | 8.6 | 93.8 | 3.5 | 2.7 | 0.04 | 27.8 | 0.04 | 73.5 | MSSA | High (26.8%) |
| 52 | 0 | 1 | 13.8 | 81.8 | 7.8 | 10.3 | 0.10 | 11.4 | 0.09 | 4.0 | MBL/ESBL | High (26.8%) |
| 53 | 0 | 1 | 5.9 | 81.3 | 3.6 | 15.0 | 0.04 | 23.4 | 0.04 | 0.5 | Other | High (26.8%) |
| 54 | 0 | 1 | 11.8 | 87.5 | 7.6 | 4.9 | 0.09 | 12.5 | 0.08 | 9.8 | MBL/ESBL | High (26.8%) |
| 55 | 0 | 1 | 14.8 | 73.5 | 14.5 | 12.0 | 0.20 | 6.1 | 0.17 | 1.7 | MBL/ESBL | High (26.8%) |
| 56 | 0 | 1 | 22.7 | 70.7 | 5.4 | 23.9 | 0.08 | 14.0 | 0.07 | 34.4 | MBL/ESBL | High (26.8%) |
| 57 | 0 | 1 | 11.9 | 89.7 | 4.0 | 6.3 | 0.04 | 23.4 | 0.04 | 28.4 | MSSA | High (26.8%) |
| 58 | 0 | 1 | 3.9 | 63.6 | 8.0 | 28.4 | 0.13 | 8.9 | 0.11 | 4.8 | Other | High (26.8%) |
| 59 | 0 | 1 | 10.6 | 83.5 | 5.3 | 11.3 | 0.06 | 16.8 | 0.06 | 12.9 | Other | High (26.8%) |
| 60 | 0 | 1 | 14.9 | 4.1 | 4.9 | 91.0 | 1.20 | 1.8 | 0.54 | 14.4 | MBL/ESBL | Low (0%) |
| 61 | 0 | 1 | 44.5 | 3.0 | 2.0 | 95.0 | 0.67 | 2.5 | 0.40 | * | MBL/ESBL | Low (0%) |
| 62 | 0 | 1 | 16.8 | 51.8 | 6.6 | 41.6 | 0.13 | 8.9 | 0.11 | 9.0 | Other | High (26.8%) |
| 63 | 0 | 1 | 5.8 | 59.0 | 7.4 | 33.5 | 0.13 | 8.9 | 0.11 | * | Other | High (26.8%) |
| 64 | 0 | 1 | 13.3 | 43.8 | 7.6 | 48.6 | 0.17 | 6.8 | 0.15 | * | Other | High (26.8%) |
| 65 | 0 | 1 | 4.1 | 95.7 | 1.6 | 2.7 | 0.02 | 60.8 | 0.02 | 21.8 | MBL/ESBL | High (26.8%) |
| 66 | 0 | 1 | 10.1 | 53.1 | 9.5 | 37.3 | 0.18 | 6.6 | 0.15 | 22.0 | Other | High (26.8%) |
| 67 | 0 | 1 | 14.1 | 89.6 | 0.3 | 10.1 | 0.00 | 298.3 | 0.00 | 0.3 | Other | High (26.8%) |
| 68 | 0 | 1 | 4.0 | 82.7 | 8.0 | 9.2 | 0.10 | 11.3 | 0.09 | * | Other | High (26.8%) |
| 69 | 0 | 1 | 8.4 | 91.6 | 4.5 | 3.9 | 0.05 | 21.3 | 0.05 | 0.2 | MBL/ESBL | High (26.8%) |
| 70 | 0 | 1 | 20.8 | 80.8 | 6.9 | 12.2 | 0.09 | 12.7 | 0.08 | * | Other | High (26.8%) |
| 71 | 0 | 1 | 5.4 | 85.2 | 0.8 | 14.0 | 0.01 | 105.8 | 0.01 | 0.4 | Other | High (26.8%) |
| 72 | 0 | 1 | 8.6 | 75.4 | 10.3 | 14.2 | 0.14 | 8.3 | 0.12 | 1.9 | Other | High (26.8%) |
| 73 | 0 | 1 | 7.6 | 91.7 | 2.1 | 6.2 | 0.02 | 44.0 | 0.02 | 8.6 | MBL/ESBL | High (26.8%) |
| 74 | 0 | 1 | 7.3 | 85.8 | 4.4 | 9.8 | 0.05 | 20.5 | 0.05 | 0.9 | MBL/ESBL | High (26.8%) |
| 75 | 0 | 1 | 10.7 | 81.6 | 7.2 | 11.1 | 0.09 | 12.3 | 0.08 | * | Other | High (26.8%) |
| 76 | 0 | 1 | 12.4 | 59.6 | 12.2 | 28.2 | 0.21 | 5.9 | 0.17 | 13.8 | MBL/ESBL | High (26.8%) |
| 77 | 0 | 1 | 1.4 | 63.0 | 18.3 | 18.7 | 0.29 | 4.4 | 0.22 | 13.8 | Other | Low (0%) |
| 78 | 0 | 1 | 17.5 | 88.9 | 5.8 | 5.3 | 0.07 | 16.3 | 0.06 | * | Other | High (26.8%) |
| 79 | 0 | 1 | 10.8 | 57.0 | 16.0 | 27.0 | 0.28 | 4.6 | 0.22 | 7.3 | Other | Low (0%) |
| 80 | 0 | 1 | 13.3 | 30.7 | 12.4 | 56.9 | 0.40 | 3.5 | 0.29 | 12.7 | Other | Low (0%) |
| 81 | 0 | 1 | 8.1 | 95.7 | 0.7 | 3.6 | 0.01 | 137.3 | 0.01 | 1.9 | Other | High (26.8%) |
| 82 | 0 | 1 | 40.7 | 90.3 | 5.7 | 4.0 | 0.06 | 16.8 | 0.06 | 35.3 | MBL/ESBL | High (26.8%) |
| 83 | 0 | 1 | 6.3 | 50.4 | 13.3 | 36.3 | 0.26 | 4.8 | 0.21 | * | Other | Low (0%) |
| 84 | 0 | 1 | 12.4 | 87.4 | 6.6 | 6.1 | 0.08 | 14.3 | 0.07 | * | Other | High (26.8%) |
| 185 | 1 | 1 | 12.5 | 83.9 | 10.0 | 6.1 | 0.12 | 9.4 | 0.11 | * | MBL/ESBL | High (26.8%) |
| 86 | 1 | 1 | 7.2 | 72.9 | 9.8 | 17.3 | 0.13 | 8.4 | 0.12 | * | Other | High (26.8%) |
| 87 | 1 | 1 | 12.5 | 81.0 | 4.1 | 14.9 | 0.05 | 20.7 | 0.05 | * | Other | High (26.8%) |
| 88 | 0 | 1 | 8.0 | 75.8 | 8.9 | 15.3 | 0.12 | 9.6 | 0.10 | * | Other | High (26.8%) |
| 89 | 1 | 1 | 5.2 | 91.8 | 4.8 | 3.4 | 0.05 | 20.1 | 0.05 | * | MBL/ESBL | High (26.8%) |
| 90 | 0 | 1 | 10.1 | 78.6 | 9.3 | 12.1 | 0.12 | 9.4 | 0.11 | 369.0 | MSSA | High (26.8%) |
| 91 | 0 | 1 | 7.8 | 84.8 | 3.5 | 11.7 | 0.04 | 25.1 | 0.04 | 94.7 | MBL/ESBL | High (26.8%) |
| 92 | 0 | 1 | 9.1 | 92.3 | 6.1 | 1.6 | 0.07 | 16.1 | 0.06 | * | Other | High (26.8%) |
| 93 | 1 | 1 | 15.3 | 87.1 | 5.9 | 7.0 | 0.07 | 15.7 | 0.06 | 218.8 | Other | High (26.8%) |
| 94 | 0 | 1 | 18.3 | 94.3 | 3.4 | 2.2 | 0.04 | 28.4 | 0.04 | 9.9 | Other | High (26.8%) |
| 95 | 0 | 1 | 9.5 | 88.9 | 6.8 | 4.2 | 0.08 | 14.0 | 0.07 | * | Other | High (26.8%) |
| 96 | 1 | 1 | 9.4 | 88.7 | 7.7 | 3.5 | 0.09 | 12.5 | 0.08 | * | Other | High (26.8%) |
| 97 | 1 | 1 | 8.1 | 76.1 | 14.4 | 9.5 | 0.19 | 6.3 | 0.16 | * | Other | High (26.8%) |
| 98 | 0 | 1 | 12.9 | 92.8 | 4.0 | 3.2 | 0.04 | 24.1 | 0.04 | * | MBL/ESBL | High (26.8%) |
| 99 | 0 | 1 | 17.7 | 87.5 | 5.2 | 7.4 | 0.06 | 18.0 | 0.06 | * | MBL/ESBL | High (26.8%) |
| 100 | 0 | 1 | 12.9 | 93.3 | 3.9 | 2.8 | 0.04 | 24.9 | 0.04 | 1.4 | Other | High (26.8%) |

Table 1 (cont’d)

| **ID^A^** | **Outcome^B^** | **Class^C^** | **WBC^D^** | **N%^E^** | **M%^F^** | **L%^G^** | **AE^H^** | **AAQ ^I^** | **BAR^J^** | **CRP^K^** | **Bacter^L^** | **Day-1 pp.^M^** |
| --- | --- | --- | --- | --- | --- | --- | --- | --- | --- | --- | --- | --- |
| 101 | 0 | 1 | 10.2 | 52.8 | 10.1 | 37.1 | 0.19 | 6.3 | 0.16 | * | MBL/ESBL | High (26.8%) |
| 102 | 0 | 1 | 3.9 | 81.1 | 8.1 | 10.8 | 0.10 | 11.0 | 0.09 | * | MBL/ESBL | High (26.8%) |
| 103 | 0 | 1 | 11.5 | 70.9 | 12.3 | 16.8 | 0.17 | 6.8 | 0.15 | * | Other | High (26.8%) |
| 104 | 0 | 1 | 10.5 | 58.7 | 13.6 | 27.7 | 0.23 | 5.3 | 0.19 | * | MBL/ESBL | High (26.8%) |
| 105 | 1 | 1 | 2.8 | 86.2 | 9.3 | 4.5 | 0.11 | 10.3 | 0.10 | * | MBL/ESBL | High (26.8%) |
| 106 | 1 | 1 | 15.4 | 90.0 | 5.8 | 4.2 | 0.06 | 16.6 | 0.06 | * | MBL/ESBL | High (26.8%) |
| 107 | 1 | 1 | 12.4 | 70.0 | 11.1 | 18.9 | 0.16 | 7.3 | 0.14 | * | Other | High (26.8%) |
| 108 | 0 | 1 | 8.5 | 64.6 | 5.2 | 30.2 | 0.08 | 13.5 | 0.07 | * | Other | High (26.8%) |
| 109 | 0 | 1 | 9.6 | 85.1 | 2.4 | 12.4 | 0.03 | 36.3 | 0.03 | * | Other | High (26.8%) |
| 110 | 0 | 1 | 11.0 | 92.9 | 4.0 | 3.1 | 0.04 | 24.1 | 0.04 | * | Other | High (26.8%) |
| 111 | 0 | 1 | 12.6 | 91.1 | 4.1 | 4.8 | 0.05 | 23.2 | 0.04 | 259 | Other | High (26.8%) |
| 112 | 0 | 1 | 13.6 | 82.4 | 2.6 | 15.1 | 0.03 | 33.0 | 0.03 | * | Other | High (26.8%) |
| 113 | 0 | 1 | 10.0 | 72.8 | 5.7 | 21.5 | 0.08 | 13.8 | 0.07 | * | MBL/ESBL | High (26.8%) |
| 114 | 0 | 1 | 10.7 | 89.2 | 4.4 | 6.5 | 0.05 | 21.5 | 0.05 | * | MBL/ESBL | High (26.8%) |
| 115 | 1 | 1 | 15.1 | 92.6 | 2.7 | 4.7 | 0.03 | 35.3 | 0.03 | 58.9 | MBL/ESBL | High (26.8%) |
| 116 | 0 | 1 | 18.5 | 84.7 | 8.8 | 6.5 | 0.10 | 10.6 | 0.09 | * | Other | High (26.8%) |
| 117 | 0 | 1 | 10.9 | 83.6 | 9.6 | 6.8 | 0.11 | 9.7 | 0.10 | * | Other | High (26.8%) |
| 118 | 0 | 1 | 9.3 | 92.9 | 4.3 | 2.8 | 0.05 | 22.5 | 0.04 | * | Other | High (26.8%) |
| 119 | 1 | 1 | 17.0 | 88.4 | 1.7 | 9.9 | 0.02 | 52.8 | 0.02 | * | Other | High (26.8%) |
| 120 | 1 | 1 | 9.1 | 82.3 | 5.9 | 11.8 | 0.07 | 14.8 | 0.07 | * | Other | High (26.8%) |
| 121 | 0 | 1 | 21.1 | 81.2 | 8.6 | 10.2 | 0.11 | 10.5 | 0.10 | * | MBL/ESBL | High (26.8%) |
| 122 | 0 | 1 | 10.2 | 60.2 | 6.1 | 33.7 | 0.10 | 10.9 | 0.09 | 11.3 | Other | High (26.8%) |
| 123 | 1 | 1 | 4.5 | 60.0 | 6.1 | 33.9 | 0.10 | 10.8 | 0.09 | * | Other | High (26.8%) |
| 124 | 1 | 1 | 10.5 | 71.5 | 10.7 | 17.8 | 0.15 | 7.7 | 0.13 | 102 | Other | High (26.8%) |
| 125 | 1 | 1 | 25.8 | 82.5 | 7.8 | 9.7 | 0.09 | 11.6 | 0.09 | * | MBL/ESBL | High (26.8%) |
| 126 | 1 | 1 | 9.8 | 79.6 | 10.1 | 10.2 | 0.13 | 8.8 | 0.11 | * | Other | High (26.8%) |
| 127 | 1 | 1 | 5.4 | 81.1 | 11.0 | 7.9 | 0.14 | 8.3 | 0.12 | * | Other | High (26.8%) |
| 128 | 1 | 1 | 9.1 | 90.4 | 5.8 | 3.8 | 0.06 | 16.6 | 0.06 | * | Other | High (26.8%) |
| 129 | 0 | 1 | 28.6 | 75.8 | 9.0 | 15.2 | 0.12 | 9.4 | 0.11 | 7.3 | Other | High (26.8%) |
| 130 | 0 | 1 | 10.1 | 79.1 | 10.6 | 10.2 | 0.13 | 8.4 | 0.12 | * | Other | High (26.8%) |
| 131 | 0 | 1 | 5.8 | 69.6 | 9.7 | 20.6 | 0.14 | 8.1 | 0.12 | * | Other | High (26.8%) |
| 132 | 0 | 1 | 11.7 | 67.4 | 13.5 | 19.1 | 0.20 | 6.0 | 0.17 | * | Other | High (26.8%) |
| 133 | 0 | 1 | 15.9 | 89.8 | 4.6 | 5.6 | 0.05 | 20.5 | 0.05 | * | Other | High (26.8%) |
| 134 | 0 | 1 | 9.9 | 89.5 | 4.3 | 6.2 | 0.05 | 21.8 | 0.05 | 23.6 | Other | High (26.8%) |
| 135 | 1 | 1 | 9.4 | 96.2 | 2.5 | 1.3 | 0.03 | 39.5 | 0.03 | 19.6 | Other | High (26.8%) |
| 136 | 0 | 1 | 22.8 | 85.9 | 6.7 | 7.4 | 0.08 | 13.8 | 0.07 | * | Other | High (26.8%) |
| 137 | 0 | 1 | 12.0 | 74.8 | 7.0 | 18.3 | 0.09 | 11.7 | 0.09 | * | Other | High (26.8%) |
| 138 | 1 | 1 | 9.9 | 86.7 | 4.0 | 9.3 | 0.05 | 22.6 | 0.04 | * | Other | High (26.8%) |
| 139 | 0 | 1 | 7.1 | 80.2 | 3.6 | 16.2 | 0.04 | 23.3 | 0.04 | * | Other | High (26.8%) |
| 140 | 1 | 1 | 27.8 | 82.6 | 7.2 | 10.2 | 0.09 | 12.4 | 0.08 | 86.1 | Other | High (26.8%) |
| 141 | 0 | 1 | 10.3 | 91.1 | 5.0 | 3.9 | 0.05 | 19.2 | 0.05 | * | Other | High (26.8%) |
| 142 | 0 | 1 | 18.2 | 84.8 | 6.0 | 9.2 | 0.07 | 15.1 | 0.07 | 22.2 | Other | High (26.8%) |
| 143 | 1 | 1 | 13.1 | 87.1 | 6.4 | 6.6 | 0.07 | 14.7 | 0.07 | * | Other | High (26.8%) |
| 144 | 0 | 1 | 9.0 | 72.4 | 23.8 | 3.7 | 0.33 | 4.0 | 0.25 | 42.5 | Other | Low (0%) |
| 145 | 0 | 1 | 19.0 | 72.7 | 8.4 | 18.9 | 0.12 | 9.6 | 0.10 | * | Other | High (26.8%) |
| 146 | 0 | 1 | 10.6 | 84.7 | 7.7 | 7.6 | 0.09 | 12.0 | 0.08 | * | Other | High (26.8%) |
| 147 | 1 | 1 | 8.3 | 82.9 | 6.2 | 10.9 | 0.07 | 14.3 | 0.07 | * | Other | High (26.8%) |
| 148 | 0 | 1 | 15.9 | 72.9 | 9.5 | 17.6 | 0.13 | 8.7 | 0.12 | * | Other | High (26.8%) |
| 149 | 0 | 1 | 18.5 | 71.8 | 7.8 | 20.4 | 0.11 | 10.2 | 0.10 | * | Other | High (26.8%) |
| 150 | 0 | 1 | 6.0 | 59.6 | 9.6 | 30.8 | 0.16 | 7.2 | 0.14 | * | Other | High (26.8%) |

Table 1 (cont’d)

| **ID^A^** | **Outcome^B^** | **Class^C^** | **WBC^D^** | **N%^E^** | **M%^F^** | **L%^G^** | **AE^H^** | **AAQ ^I^** | **BAR^J^** | **CRP^K^** | **Bacter^L^** | **Day-1 pp.^M^** |
| --- | --- | --- | --- | --- | --- | --- | --- | --- | --- | --- | --- | --- |
| 151 | 0 | 1 | 11.8 | 86.0 | 3.1 | 10.9 | 0.04 | 28.7 | 0.03 | * | MBL/ESBL | High (26.8%) |
| 152 | 1 | 1 | 10.2 | 91.8 | 5.2 | 3.0 | 0.06 | 18.8 | 0.05 | * | MBL/ESBL | High (26.8%) |
| 153 | 0 | 1 | 2.2 | 88.1 | 3.4 | 8.5 | 0.04 | 26.9 | 0.04 | * | Other | High (26.8%) |
| 154 | 0 | 1 | 13.7 | 80.8 | 6.3 | 12.9 | 0.08 | 13.9 | 0.07 | * | MBL/ESBL | High (26.8%) |
| 155 | 0 | 1 | 7.9 | 77.1 | 8.7 | 14.2 | 0.11 | 9.8 | 0.10 | 127.50 | Other | High (26.8%) |
| 156 | 0 | 1 | 6.9 | 82.7 | 6.5 | 10.9 | 0.08 | 13.8 | 0.07 | 0.10 | Other | High (26.8%) |
| 157 | 0 | 1 | 7.5 | 87.0 | 5.9 | 7.1 | 0.07 | 15.8 | 0.06 | 84.70 | Other | High (26.8%) |
| 158 | 0 | 1 | 12.1 | 83.2 | 2.7 | 14.1 | 0.03 | 31.4 | 0.03 | 189.40 | MSSA | High (26.8%) |
| 159 | 0 | 1 | 11.6 | 70.4 | 14.8 | 14.8 | 0.21 | 5.7 | 0.17 | 74.10 | MBL/ESBL | High (26.8%) |
| 160 | 0 | 1 | 12.6 | 7.6 | 13.2 | 79.2 | 1.72 | 1.6 | 0.63 | 194.00 | MBL/ESBL | Low (0%) |
| 161 | 0 | 1 | 7.9 | 41.4 | 9.0 | 49.6 | 0.22 | 5.6 | 0.18 | 3.00 | MBL/ESBL | High (26.8%) |
| 162 | 0 | 1 | 16.7 | 94.9 | 2.9 | 2.2 | 0.03 | 33.7 | 0.03 | 6.00 | MBL/ESBL | High (26.8%) |
| 163 | 0 | 1 | 14.1 | 75.3 | 5.8 | 18.9 | 0.08 | 14.0 | 0.07 | 195.30 | MSSA | High (26.8%) |
| 164 | 0 | 1 | 11.0 | 75.3 | 9.5 | 15.2 | 0.13 | 8.9 | 0.11 | 97.50 | MBL/ESBL | High (26.8%) |
| 165 | 0 | 1 | 18.0 | 74.5 | 15.8 | 9.7 | 0.21 | 5.7 | 0.17 | 48.30 | MBL/ESBL | High (26.8%) |
| 166 | 1 | 1 | 4.4 | 86.9 | 4.0 | 9.1 | 0.05 | 22.7 | 0.04 | * | MBL/ESBL | High (26.8%) |
| 167 | 1 | 1 | 13.6 | 89.0 | 5.3 | 5.7 | 0.06 | 17.8 | 0.06 | * | MSSA | High (26.8%) |
| 168 | 1 | 1 | 6.5 | 83.8 | 6.6 | 9.7 | 0.08 | 13.8 | 0.07 | 1.60 | Other | High (26.8%) |
| 169 | 0 | 1 | 22.4 | 78.1 | 2.0 | 19.9 | 0.03 | 39.8 | 0.03 | 1.50 | Other | High (26.8%) |
| 170 | 0 | 1 | 7.4 | 68.1 | 9.3 | 22.6 | 0.14 | 8.3 | 0.12 | 1.20 | MBL/ESBL | High (26.8%) |
| 171 | 1 | 1 | 11.2 | 90.2 | 5.6 | 4.2 | 0.06 | 17.2 | 0.06 | 132.40 | Other | High (26.8%) |
| 172 | 0 | 1 | 18.8 | 89.0 | 5.4 | 5.5 | 0.06 | 17.4 | 0.06 | 351.90 | MBL/ESBL | High (26.8%) |
| 173 | 0 | 1 | 20.2 | 77.4 | 9.9 | 12.7 | 0.13 | 8.8 | 0.11 | 67.70 | MBL/ESBL | High (26.8%) |
| 174 | 1 | 1 | 3.1 | 96.8 | 1.0 | 2.2 | 0.01 | 96.4 | 0.01 | 355.50 | Other | High (26.8%) |
| 175 | 0 | 1 | 4.9 | 77.5 | 12.1 | 10.4 | 0.16 | 7.4 | 0.13 | 7.50 | Other | High (26.8%) |
| 176 | 0 | 1 | 8.9 | 73.7 | 8.0 | 18.4 | 0.11 | 10.2 | 0.10 | 64.40 | Other | High (26.8%) |
| 177 | 0 | 1 | 8.0 | 77.7 | 6.8 | 15.5 | 0.09 | 12.4 | 0.08 | 1.50 | Other | High (26.8%) |
| 178 | 0 | 1 | 22.0 | 94.0 | 3.9 | 2.1 | 0.04 | 25.1 | 0.04 | * | MBL/ESBL | High (26.8%) |
| 179 | 1 | 1 | 19.8 | 92.2 | 3.7 | 4.0 | 0.04 | 25.8 | 0.04 | 244.60 | Other | High (26.8%) |
| 180 | 1 | 1 | 7.5 | 80.4 | 6.2 | 13.4 | 0.08 | 13.9 | 0.07 | 84.30 | Other | High (26.8%) |
| 181 | 0 | 1 | 11.4 | 82.1 | 4.3 | 13.7 | 0.05 | 20.3 | 0.05 | 170.90 | MBL/ESBL | High (26.8%) |
| 182 | 1 | 1 | 6.9 | 82.6 | 4.6 | 12.7 | 0.06 | 18.9 | 0.05 | 195.90 | Other | High (26.8%) |
| 183 | 1 | 1 | 3.0 | 85.4 | 4.0 | 10.6 | 0.05 | 22.1 | 0.05 | 75.50 | MBL/ESBL | High (26.8%) |
| 184 | 1 | 1 | 28.2 | 87.1 | 3.2 | 9.6 | 0.04 | 27.9 | 0.04 | 24.80 | Other | High (26.8%) |
| 185 | 1 | 1 | 8.1 | 83.0 | 6.4 | 10.5 | 0.08 | 13.9 | 0.07 | 147.20 | MBL/ESBL | High (26.8%) |
| 186 | 1 | 1 | 16.9 | 80.3 | 5.4 | 14.2 | 0.07 | 15.8 | 0.06 | 239.50 | MBL/ESBL | High (26.8%) |
| 187 | 1 | 1 | 12.3 | 71.9 | 9.7 | 18.4 | 0.14 | 8.4 | 0.12 | 114.50 | Other | High (26.8%) |
| 188 | 0 | 1 | 18.4 | 76.7 | 6.7 | 16.5 | 0.09 | 12.4 | 0.08 | 4.80 | Other | High (26.8%) |
| 189 | 0 | 1 | 33.4 | 75.7 | 5.9 | 18.4 | 0.08 | 13.8 | 0.07 | 25.90 | MRSA | High (26.8%) |
| 190 | 0 | 1 | 11.9 | 74.6 | 9.4 | 16.0 | 0.13 | 8.9 | 0.11 | 12.00 | MBL/ESBL | High (26.8%) |
| 191 | 0 | 1 | 17.5 | 71.4 | 8.8 | 19.7 | 0.12 | 9.1 | 0.11 | 117.80 | Other | High (26.8%) |
| 192 | 0 | 1 | 17.2 | 75.5 | 6.1 | 18.4 | 0.08 | 13.3 | 0.08 | 13.50 | Other | High (26.8%) |
| 193 | 0 | 1 | 16.8 | 75.8 | 6.4 | 17.9 | 0.08 | 12.9 | 0.08 | 206.00 | MBL/ESBL | High (26.8%) |
| 194 | 1 | 1 | 21.3 | 91.7 | 4.9 | 3.4 | 0.05 | 19.7 | 0.05 | 313.60 | Other | High (26.8%) |
| 195 | 1 | 1 | 15.4 | 81.8 | 8.5 | 9.8 | 0.10 | 10.7 | 0.09 | 207.50 | Other | High (26.8%) |
| 196 | 1 | 1 | 7.2 | 96.2 | 1.0 | 2.8 | 0.01 | 95.4 | 0.01 | 8.80 | Other | High (26.8%) |
| 197 | 1 | 1 | 19.3 | 95.9 | 2.0 | 2.1 | 0.02 | 48.9 | 0.02 | 235.40 | MBL/ESBL | High (26.8%) |
| 198 | 0 | 1 | 13.1 | 78.5 | 10.8 | 10.8 | 0.14 | 8.3 | 0.12 | 106.60 | Other | High (26.8%) |
| 199 | 0 | 1 | 9.2 | 71.4 | 7.0 | 21.6 | 0.10 | 11.2 | 0.09 | 30.70 | Other | High (26.8%) |
| 200 | 1 | 1 | 5.7 | 52.1 | 8.5 | 39.4 | 0.16 | 7.1 | 0.14 | * | MBL/ESBL | High (26.8%) |

Table 1 (cont’d)

| **ID^A^** | **Outcome^B^** | **Class^C^** | **WBC^D^** | **N%^E^** | **M%^F^** | **L%^G^** | **AE^H^** | **AAQ ^I^** | **BAR^J^** | **CRP^K^** | **Bacter^L^** | **Day-1 pp.^M^** |
| --- | --- | --- | --- | --- | --- | --- | --- | --- | --- | --- | --- | --- |
| 201 | 1 | 1 | 3.5 | 66.8 | 15.3 | 3.5 | 0.23 | 5.4 | 0.19 | 196.90 | Other | High (26.8%) |
| 202 | 0 | 1 | 7.0 | 79.2 | 6.5 | 7.0 | 0.08 | 13.1 | 0.08 | * | Other | High (26.8%) |
| 203 | 0 | 1 | 12.0 | 74.5 | 12.1 | 12.0 | 0.16 | 7.2 | 0.14 | 131.30 | MBL/ESBL | High (26.8%) |
| 204 | 0 | 1 | 10.8 | 82.7 | 0.6 | 10.8 | 0.01 | 138.5 | 0.01 | * | Other | High (26.8%) |
| 205 | 0 | 1 | 19.2 | 83.9 | 7.7 | 19.2 | 0.09 | 11.8 | 0.08 | 224.00 | MSSA | High (26.8%) |
| 206 | 1 | 1 | 10.2 | 83.8 | 5.3 | 10.2 | 0.06 | 16.8 | 0.06 | 145.60 | MBL/ESBL | High (26.8%) |
| 207 | 1 | 1 | 7.3 | 82.9 | 4.1 | 7.3 | 0.05 | 21.0 | 0.05 | 189.50 | MBL/ESBL | High (26.8%) |
| 208 | 0 | 1 | 7.3 | 71.6 | 7.8 | 7.3 | 0.11 | 10.2 | 0.10 | 6.90 | Other | High (26.8%) |
| 209 | 0 | 1 | 8.4 | 77.7 | 8.0 | 8.4 | 0.10 | 10.7 | 0.09 | * | MBL/ESBL | High (26.8%) |
| 210 | 0 | 1 | 6.9 | 67.4 | 4.0 | 6.9 | 0.06 | 17.8 | 0.06 | 66.30 | Other | High (26.8%) |
| 211 | 0 | 1 | 23.8 | 86.2 | 2.8 | 23.8 | 0.03 | 31.7 | 0.03 | 11.00 | Other | High (26.8%) |
| 212 | 0 | 1 | 40.6 | 73.9 | 7.8 | 40.6 | 0.11 | 10.5 | 0.10 | 134.30 | MBL/ESBL | High (26.8%) |
| 213 | 0 | 1 | 11.8 | 81.2 | 7.6 | 11.8 | 0.09 | 11.7 | 0.09 | 35.50 | Other | High (26.8%) |
| 214 | 0 | 1 | 7.1 | 78.6 | 6.0 | 7.1 | 0.08 | 14.1 | 0.07 | 84.10 | Other | High (26.8%) |
| 215 | 0 | 1 | 16.9 | 82.3 | 7.3 | 16.9 | 0.09 | 12.3 | 0.08 | 339.50 | Other | High (26.8%) |
| 216 | 0 | 1 | 12.4 | 71.5 | 12.2 | 12.4 | 0.17 | 6.9 | 0.15 | 92.20 | Other | High (26.8%) |
| 217 | 0 | 1 | 8.5 | 69.9 | 9.6 | 8.5 | 0.14 | 8.2 | 0.12 | * | MBL/ESBL | High (26.8%) |
| 218 | 0 | 1 | 9.0 | 91.7 | 4.7 | 9.0 | 0.05 | 20.5 | 0.05 | 74.80 | MBL/ESBL | High (26.8%) |
| 219 | 0 | 1 | 15.0 | 75.4 | 6.1 | 15.0 | 0.08 | 13.4 | 0.07 | 253.10 | Other | High (26.8%) |
| 220 | 0 | 1 | 5.5 | 93.5 | 2.2 | 5.5 | 0.02 | 43.5 | 0.02 | * | MRSA | High (26.8%) |
| 221 | 1 | 1 | 10.8 | 80.0 | 8.1 | 10.8 | 0.10 | 10.9 | 0.09 | * | MBL/ESBL | High (26.8%) |
| 222 | 1 | 1 | 14.9 | 82.0 | 10.2 | 14.9 | 0.12 | 9.1 | 0.11 | 83.50 | MBL/ESBL | High (26.8%) |
| 223 | 1 | 1 | 3.3 | 68.4 | 11.0 | 3.3 | 0.16 | 7.2 | 0.14 | 106.20 | MBL/ESBL | High (26.8%) |
| 224 | 1 | 1 | 9.6 | 61.3 | 13.8 | 9.6 | 0.23 | 5.4 | 0.18 | 9.00 | Other | High (26.8%) |
| 225 | 1 | 1 | 4.4 | 66.4 | 6.6 | 4.4 | 0.10 | 11.0 | 0.09 | 70.90 | MBL/ESBL | High (26.8%) |
| 226 | 0 | 1 | 13.3 | 93.1 | 3.5 | 13.3 | 0.04 | 27.5 | 0.04 | 8.40 | Other | High (26.8%) |
| 227 | 1 | 1 | 11.5 | 93.6 | 2.7 | 11.5 | 0.03 | 35.4 | 0.03 | 67.40 | Other | High (26.8%) |
| 228 | 0 | 1 | 11.6 | 79.1 | 8.9 | 11.6 | 0.11 | 9.9 | 0.10 | 322.70 | Other | High (26.8%) |
| 229 | 0 | 1 | 14.7 | 75.6 | 6.2 | 14.7 | 0.08 | 13.1 | 0.08 | 139.10 | MBL/ESBL | High (26.8%) |
| 230 | 0 | 1 | 12.5 | 88.3 | 2.8 | 12.5 | 0.03 | 32.5 | 0.03 | 11.50 | MBL/ESBL | High (26.8%) |
| 231 | 0 | 1 | 6.8 | 75.8 | 5.8 | 6.8 | 0.08 | 14.1 | 0.07 | 1.40 | Other | High (26.8%) |
| 232 | 1 | 1 | 11.2 | 88.0 | 5.2 | 11.2 | 0.06 | 18.0 | 0.06 | 260.50 | Other | High (26.8%) |
| 233 | 0 | 1 | 15.9 | 83.6 | 8.8 | 15.9 | 0.10 | 10.5 | 0.09 | 27.80 | Other | High (26.8%) |
| 234 | 0 | 1 | 15.4 | 87.3 | 5.5 | 15.4 | 0.06 | 17.0 | 0.06 | 324.10 | Other | High (26.8%) |
| 235 | 0 | 1 | 10.7 | 81.1 | 6.5 | 10.7 | 0.08 | 13.5 | 0.07 | 150.30 | Other | High (26.8%) |
| 236 | 0 | 1 | 10.1 | 89.8 | 6.0 | 10.1 | 0.07 | 15.9 | 0.06 | 52.10 | MBL/ESBL | High (26.8%) |
| 237 | 1 | 1 | 18.9 | 89.9 | 4.9 | 18.9 | 0.05 | 19.3 | 0.05 | 29.70 | Other | High (26.8%) |
| 238 | 0 | 1 | 9.6 | 93.1 | 0.7 | 9.6 | 0.01 | 133.6 | 0.01 | 232.20 | Other | High (26.8%) |
| 239 | 0 | 1 | 4.7 | 69.0 | 6.7 | 4.7 | 0.10 | 11.3 | 0.09 | 49.20 | Other | High (26.8%) |
| 240 | 0 | 1 | 15.3 | 87.1 | 8.2 | 15.3 | 0.09 | 11.6 | 0.09 | * | Other | High (26.8%) |
| 241 | 0 | 1 | 5.1 | 72.9 | 7.1 | 5.1 | 0.10 | 11.2 | 0.09 | 100.80 | Other | High (26.8%) |
| 242 | 1 | 1 | 14.3 | 87.9 | 2.5 | 14.3 | 0.03 | 36.1 | 0.03 | * | Other | High (26.8%) |
| 243 | 1 | 1 | 12.9 | 85.3 | 3.8 | 12.9 | 0.04 | 23.4 | 0.04 | 305.90 | Other | High (26.8%) |
| 244 | 0 | 1 | 14.3 | 75.2 | 8.0 | 14.3 | 0.11 | 10.5 | 0.10 | 144.30 | MBL/ESBL | High (26.8%) |
| 245 | 0 | 1 | 10.8 | 88.3 | 7.7 | 10.8 | 0.09 | 12.4 | 0.08 | 33.20 | Other | High (26.8%) |
| 246 | 1 | 1 | 17.1 | 52.7 | 1.8 | 17.1 | 0.03 | 31.0 | 0.03 | 48.90 | MBL/ESBL | High (26.8%) |
| 247 | 1 | 1 | 4.0 | 48.9 | 1.1 | 4.0 | 0.02 | 45.5 | 0.02 | 9.50 | Other | High (26.8%) |
| 248 | 0 | 1 | 6.3 | 72.0 | 11.1 | 6.3 | 0.15 | 7.5 | 0.13 | 3.00 | MBL/ESBL | High (26.8%) |
| 249 | 0 | 1 | 8.2 | 82.4 | 6.6 | 8.2 | 0.08 | 13.4 | 0.07 | 172.40 | Other | High (26.8%) |
| 250 | 0 | 1 | 13.3 | 80.4 | 6.8 | 13.3 | 0.08 | 12.8 | 0.08 | 28.20 | MBL/ESBL | High (26.8%) |

Table 1 (cont’d)

| **ID^A^** | **Outcome^B^** | **Class^C^** | **WBC^D^** | **N%^E^** | **M%^F^** | **L%^G^** | **AE^H^** | **AAQ ^I^** | **BAR^J^** | **CRP^K^** | **Bacter^L^** | **Day-1 pp.^M^** |
| --- | --- | --- | --- | --- | --- | --- | --- | --- | --- | --- | --- | --- |
| 251 | 0 | 1 | 9.5 | 82.6 | 3.8 | 13.6 | 0.05 | 5.4 | 0.04 | 57.30 | Other | High (26.8%) |
| 252 | 0 | 1 | 6.9 | 82.5 | 7.8 | 9.7 | 0.09 | 13.1 | 0.09 | 61.60 | Other | High (26.8%) |
| 253 | 0 | 1 | 3.5 | 87.0 | 5.5 | 7.5 | 0.06 | 7.2 | 0.06 | 163.30 | Other | High (26.8%) |
| 254 | 0 | 1 | 14.6 | 71.5 | 8.3 | 20.2 | 0.12 | 138.5 | 0.10 | 147.20 | Other | High (26.8%) |
| 255 | 1 | 1 | 17.7 | 94.7 | 2.0 | 3.3 | 0.02 | 11.8 | 0.02 | 83.20 | MSSA | High (26.8%) |
| 256 | 0 | 1 | 6.9 | 80.9 | 6.9 | 12.2 | 0.09 | 16.8 | 0.08 | 69.30 | Other | High (26.8%) |
| 257 | 0 | 1 | 7.2 | 66.9 | 9.8 | 23.3 | 0.15 | 21.0 | 0.13 | * | MBL/ESBL | High (26.8%) |
| 258 | 0 | 1 | 12.8 | 94.1 | 1.4 | 4.5 | 0.01 | 10.2 | 0.01 | 141.00 | MBL/ESBL | High (26.8%) |
| 259 | 0 | 1 | 6.4 | 82.9 | 3.1 | 14.0 | 0.04 | 10.7 | 0.04 | 106.50 | Other | High (26.8%) |
| 260 | 0 | 1 | 11.8 | 76.1 | 4.6 | 19.3 | 0.06 | 17.8 | 0.06 | 93.20 | MBL/ESBL | High (26.8%) |
| 261 | 0 | 1 | 8.9 | 95.0 | 1.3 | 3.7 | 0.01 | 31.7 | 0.01 | 3.60 | Other | High (26.8%) |
| 262 | 0 | 1 | 15.1 | 81.6 | 4.5 | 13.9 | 0.06 | 10.5 | 0.05 | 18.30 | Other | High (26.8%) |
| 263 | 0 | 1 | 4.6 | 83.2 | 4.9 | 11.8 | 0.06 | 11.7 | 0.06 | 49.40 | Other | High (26.8%) |
| 264 | 0 | 1 | 4.4 | 40.2 | 11.3 | 48.5 | 0.28 | 14.1 | 0.22 | 14.00 | Other | High (26.8%) |
| 265 | 0 | 1 | 4.6 | 51.8 | 6.7 | 41.5 | 0.13 | 12.3 | 0.11 | 35.20 | Other | High (26.8%) |
| 266 | 0 | 1 | 16.6 | 82.9 | 9.2 | 7.9 | 0.11 | 6.9 | 0.10 | * | Other | High (26.8%) |
| 267 | 0 | 1 | 11.6 | 84.8 | 6.9 | 8.3 | 0.08 | 8.2 | 0.08 | * | Other | High (26.8%) |
| 268 | 0 | 1 | 4.3 | 88.3 | 4.1 | 7.6 | 0.05 | 20.5 | 0.04 | * | MRSA | High (26.8%) |
| 269 | 1 | 1 | 23.5 | 95.8 | 0.9 | 3.3 | 0.01 | 13.4 | 0.01 | * | MBL/ESBL | High (26.8%) |
| 270 | 0 | 1 | 7.6 | 86.9 | 3.7 | 9.4 | 0.04 | 43.5 | 0.04 | 69.60 | MSSA | High (26.8%) |
| 271 | 0 | 1 | 19.3 | 84.1 | 8.6 | 7.3 | 0.10 | 10.9 | 0.09 | 0.50 | Other | High (26.8%) |
| 272 | 0 | 1 | 26.5 | 97.5 | 1.5 | 1.0 | 0.02 | 9.1 | 0.02 | * | MBL/ESBL | High (26.8%) |
| 273 | 0 | 1 | 9.4 | 86.7 | 3.4 | 9.8 | 0.04 | 7.2 | 0.04 | 124.10 | Other | High (26.8%) |
| 274 | 0 | 1 | 11.8 | 74.1 | 9.5 | 16.4 | 0.13 | 5.4 | 0.11 | * | Other | High (26.8%) |
| 275 | 0 | 1 | 14.1 | 62.2 | 7.3 | 30.5 | 0.12 | 11.0 | 0.10 | * | Other | High (26.8%) |
| 276 | 0 | 1 | 13.4 | 62.9 | 7.2 | 29.9 | 0.11 | 27.5 | 0.10 | 184.90 | MBL/ESBL | High (26.8%) |
| 277 | 0 | 1 | 8.5 | 91.1 | 3.1 | 5.8 | 0.03 | 35.4 | 0.03 | 164.40 | Other | High (26.8%) |
| 278 | 0 | 1 | 11.2 | 87.5 | 3.5 | 9.0 | 0.04 | 9.9 | 0.04 | 139.80 | MBL/ESBL | High (26.8%) |
| 279 | 0 | 1 | 3.6 | 90.1 | 1.8 | 8.1 | 0.02 | 13.1 | 0.02 | 38.60 | MBL/ESBL | High (26.8%) |
| 280 | 0 | 1 | 11.4 | 72.5 | 4.2 | 23.3 | 0.06 | 32.5 | 0.05 | 112.60 | MBL/ESBL | High (26.8%) |
| 281 | 0 | 1 | 11.0 | 62.5 | 13.8 | 23.7 | 0.22 | 14.1 | 0.18 | 111.00 | Other | High (26.8%) |
| 282 | 1 | 1 | 16.0 | 87.8 | 2.1 | 10.1 | 0.02 | 18.0 | 0.02 | 11.60 | Other | High (26.8%) |
| 283 | 0 | 1 | 9.8 | 91.7 | 3.8 | 4.5 | 0.04 | 10.5 | 0.04 | 4.50 | Other | High (26.8%) |
| 284 | 0 | 1 | 12.6 | 61.6 | 25.8 | 12.7 | 0.42 | 17.0 | 0.30 | * | Other | Low (0%) |
| 285 | 1 | 1 | 10.4 | 85.2 | 7.6 | 7.1 | 0.09 | 13.5 | 0.08 | 33.55 | Other | High (26.8%) |
| 286 | 1 | 1 | 9.7 | 82.2 | 8.1 | 9.6 | 0.10 | 15.9 | 0.09 | 0.52 | Other | High (26.8%) |
| 287 | 0 | 2 | 10.1 | 80.2 | 10.0 | 9.7 | 0.13 | 19.3 | 0.11 | 10.90 | No isolation | High (26.8%) |
| 288 | 0 | 2 | 17.5 | 86.0 | 7.4 | 6.5 | 0.09 | 133.6 | 0.08 | 0.33 | No isolation | High (26.8%) |
| 289 | 0 | 2 | 16.7 | 68.2 | 6.8 | 25.0 | 0.10 | 11.3 | 0.09 | 7.86 | No isolation | High (26.8%) |
| 290 | 0 | 2 | 2.1 | 82.9 | 4.2 | 13.0 | 0.05 | 11.6 | 0.05 | 0.76 | No isolation | High (26.8%) |
| 291 | 0 | 2 | 4.3 | 89.3 | 4.2 | 6.5 | 0.05 | 11.2 | 0.05 | 6.82 | No isolation | High (26.8%) |
| 292 | 0 | 2 | 6.0 | 66.0 | 10.9 | 23.0 | 0.17 | 36.1 | 0.14 | 0.25 | No isolation | High (26.8%) |
| 293 | 0 | 2 | 7.1 | 45.3 | 34.0 | 20.7 | 0.75 | 23.4 | 0.43 | * | No isolation | Low (0%) |
| 294 | 0 | 2 | 0.0 | 73.0 | 19.9 | 7.1 | 0.27 | 10.5 | 0.21 | * | No isolation | Low (0%) |
| 295 | 0 | 2 | 2.0 | 66.6 | 16.7 | 16.7 | 0.25 | 12.4 | 0.20 | 1.07 | No isolation | High (26.8%) |
| 296 | 0 | 2 | 38.1 | 97.3 | 1.4 | 1.3 | 0.01 | 31.0 | 0.01 | 28.62 | No isolation | High (26.8%) |
| 297 | 0 | 2 | 6.7 | 67.8 | 23.5 | 8.7 | 0.35 | 45.5 | 0.26 | * | No isolation | Low (0%) |
| 298 | 0 | 2 | 5.7 | 74.8 | 8.1 | 17.1 | 0.11 | 7.5 | 0.10 | 0.11 | No isolation | High (26.8%) |
| 299 | 0 | 2 | 9.2 | 75.9 | 8.2 | 15.9 | 0.11 | 13.4 | 0.10 | * | No isolation | High (26.8%) |
| 300 | 0 | 2 | 11.3 | 64.6 | 12.7 | 22.6 | 0.20 | 12.8 | 0.16 | 18.17 | No isolation | High (26.8%) |

Table 1 (cont’d)

| **ID^A^** | **Outcome^B^** | **Class^C^** | **WBC^D^** | **N%^E^** | **M%^F^** | **L%^G^** | **AE^H^** | **AAQ ^I^** | **BAR^J^** | **CRP^K^** | **Bacter^L^** | **Day-1 pp.^M^** |
| --- | --- | --- | --- | --- | --- | --- | --- | --- | --- | --- | --- | --- |
| 301 | 0 | 2 | 8.6 | 85.9 | 6.3 | 7.8 | 0.07 | 14.6 | 0.07 | 2.84 | No isolation | High (26.8%) |
| 302 | 0 | 2 | 12.5 | 81.5 | 8.4 | 10.1 | 0.10 | 10.7 | 0.09 | 0.14 | No isolation | High (26.8%) |
| 303 | 0 | 2 | 9.2 | 76.4 | 7.1 | 16.5 | 0.09 | 11.8 | 0.08 | 21.55 | No isolation | High (26.8%) |
| 304 | 1 | 2 | 9.4 | 87.8 | 1.4 | 10.8 | 0.02 | 62.1 | 0.02 | 8.27 | No isolation | High (26.8%) |
| 305 | 0 | 2 | 11.1 | 67.5 | 10.9 | 21.6 | 0.16 | 7.2 | 0.14 | * | No isolation | High (26.8%) |
| 306 | 0 | 2 | 10.8 | 88.5 | 4.0 | 7.5 | 0.05 | 23.1 | 0.04 | 3.50 | No isolation | High (26.8%) |
| 307 | 0 | 2 | 11.9 | 14.7 | 22.2 | 63.2 | 1.51 | 1.7 | 0.60 | 1.10 | No isolation | Low (0%) |
| 308 | 0 | 2 | 3.8 | 69.5 | 10.3 | 20.2 | 0.15 | 7.8 | 0.13 | 32.60 | No isolation | High (26.8%) |
| 309 | 0 | 2 | 18.4 | 76.0 | 6.9 | 17.1 | 0.09 | 12.1 | 0.08 | 44.20 | No isolation | High (26.8%) |
| 310 | 0 | 2 | 11.0 | 95.0 | 0.6 | 4.4 | 0.01 | 158.5 | 0.01 | 9.94 | No isolation | High (26.8%) |
| 311 | 0 | 2 | 17.3 | 71.5 | 14.8 | 13.7 | 0.21 | 5.8 | 0.17 | 2.55 | No isolation | High (26.8%) |
| 312 | 1 | 2 | 5.7 | 48.1 | 9.6 | 42.4 | 0.20 | 6.0 | 0.17 | 1.89 | No isolation | High (26.8%) |
| 313 | 0 | 2 | 6.3 | 81.2 | 10.8 | 8.0 | 0.13 | 8.5 | 0.12 | 7.13 | No isolation | High (26.8%) |
| 314 | 0 | 2 | 7.8 | 69.5 | 11.4 | 19.1 | 0.16 | 7.1 | 0.14 | 4.70 | No isolation | High (26.8%) |
| 315 | 0 | 2 | 2.5 | 77.8 | 13.2 | 9.0 | 0.17 | 6.9 | 0.15 | * | No isolation | High (26.8%) |
| 316 | 0 | 2 | 25.1 | 87.4 | 5.3 | 7.3 | 0.06 | 17.5 | 0.06 | 44.14 | No isolation | High (26.8%) |
| 317 | 0 | 2 | 3.2 | 73.1 | 13.1 | 13.9 | 0.18 | 6.6 | 0.15 | * | No isolation | High (26.8%) |
| 318 | 1 | 2 | 13.5 | 61.2 | 8.3 | 30.5 | 0.14 | 8.3 | 0.12 | 12.80 | No isolation | High (26.8%) |
| 319 | 0 | 2 | 0.3 | 89.6 | 8.5 | 1.9 | 0.09 | 11.6 | 0.09 | 8.05 | No isolation | High (26.8%) |
| 320 | 0 | 2 | 19.6 | 80.7 | 8.8 | 10.5 | 0.11 | 10.2 | 0.10 | 9.15 | No isolation | High (26.8%) |
| 321 | 0 | 2 | 11.0 | 85.4 | 7.7 | 6.9 | 0.09 | 12.1 | 0.08 | 12.90 | No isolation | High (26.8%) |
| 322 | 0 | 2 | 10.7 | 90.4 | 6.5 | 3.1 | 0.07 | 14.9 | 0.07 | * | No isolation | High (26.8%) |
| 323 | 0 | 2 | 25.3 | 61.3 | 11.1 | 27.6 | 0.18 | 6.5 | 0.15 | 2.57 | No isolation | High (26.8%) |
| 324 | 0 | 2 | 9.9 | 92.4 | 5.0 | 2.6 | 0.05 | 19.4 | 0.05 | 19.02 | No isolation | High (26.8%) |
| 325 | 0 | 2 | 17.0 | 75.2 | 10.1 | 14.7 | 0.13 | 8.4 | 0.12 | 12.10 | No isolation | High (26.8%) |
| 326 | 0 | 2 | 19.1 | 72.9 | 8.2 | 18.9 | 0.11 | 9.9 | 0.10 | * | No isolation | High (26.8%) |
| 327 | 0 | 2 | 18.0 | 78.9 | 6.3 | 14.8 | 0.08 | 13.5 | 0.07 | 7.96 | No isolation | High (26.8%) |
| 328 | 0 | 2 | 7.9 | 54.6 | 8.5 | 36.9 | 0.16 | 7.4 | 0.13 | 1.11 | No isolation | High (26.8%) |
| 329 | 0 | 2 | 9.9 | 88.8 | 6.1 | 5.1 | 0.07 | 15.5 | 0.06 | 0.78 | No isolation | High (26.8%) |

Glossary

**A:** Patient identification number

**B**: Outcome (1: non-survivor; 0: survivor)

**C**: Class (1: presumed septic; 2: presumed non-septic)

**D**: White blood cell count (thousands/milliliter)

**E**: Neutrophil percentage

**F**: Monocyte percentage

**G**: Lymphocyte percentage

**H-J**: computer-derived complex, leukocyte related indicators

**K**: C-Reactive protein

**L**: Bacteriological characterization (MRSA: methicillin-resistant *S. aureus*, MSSA: methicillin-susceptible *S. aureus*, MBL/ESBL: metallo beta-lactamase and/or extended-spectrum beta-lactamase)

**M**: Day-1 personalized patient partitioning: high risk (26.8% mortality or 84/313), low risk (0% mortality or 0/16).
