# Supplementary material for: Personalized, disease-stage specific, rapid identification of immunosuppression in sepsis
Source: Front Immunol. 2024 Oct 29;15:1430972. doi: 10.3389/fimmu.2024.1430972 (PMC11558526; doi:10.3389/fimmu.2024.1430972)
Supplement: Supplementary file 2 [file Table2.docx]

**Table 2. Similarities and differences between statistical and biomedical significance**

| Variable | N | Median | *P* value | *Overlapping*  *data intervals* |
| --- | --- | --- | --- | --- |
| Early vs. late immuno-suppression |  | |  |  |
| Neutrophil % early immuno-suppression | 11 | 57.7 | **<0.01** | yes |
| Neutrophil % late immuno-suppression | 9 | 86.4 |  |  |
| Monocyte % early immuno-suppression | 11 | 21.2 | **<0.01** | **yes** |
| Monocyte % late immuno-suppression | 9 | 5.4 |  |  |
| Lymphocyte % early immuno-suppression | 11 | 18.1 | >0.05 | **yes** |
| Lymphocyte % late immuno-suppression | 9 | 6.7 |  |  |
| Early immuno-competence vs. early immuno-suppression |  | |  |  |
| Neutrophil % early immuno-competence | 26 | 3.1 | **<0.01** | **yes** |
| Neutrophil % early immuno-suppression | 11 | 57.7 |  |  |
| Monocyte % early immuno-competence | 26 | 17.3 | >0.05 | **yes** |
| Monocyte % early immuno-suppression | 11 | 21.2 |  |  |
| Lymphocyte % early immuno-competence | 26 | 79.7 | **<0.01** | **yes** |
| Lymphocyte % early immuno-suppression | 11 | 18.1 |  |  |
| Early immuno-competence vs. early inflammation |  | |  |  |
| Neutrophil % early immuno-competence | 26 | 3.1 | **<0.01** | no |
| Neutrophil % early early inflammation | 76 | 95.7 |  |  |
| Monocyte % early immuno-competence | 26 | 17.3 | **<0.01** | **yes** |
| Monocyte % early early inflammation | 76 | .6 |  |  |
| Lymphocyte % early immuno-competence | 26 | 79.7 | **<0.01** | **yes** |
| Lymphocyte % early early inflammation | 76 | 3.6 |  |  |
| *BAR*-related comparisons |  |  |  |  |
| *BAR* early immuno-suppression | 11 | .3 | **<0.01** | no |
| *BAR* late immuno-suppression | 9 | .06 |  |  |
| *BAR* early immuno-competence | 26 | .84 | **<0.01** | no |
| *BAR* late immuno-suppression | 9 | .06 |  |  |
| *BAR* early immuno-competence | 26 | .84 | **<0.01** | no |
| *BAR* early inflammation | 76 | .01 |  |  |
